# The psychosis metabolic risk calculator (PsyMetRiC) for young people with psychosis: International external validation and site-specific recalibration in two independent European samples

**DOI:** 10.1016/j.lanepe.2022.100493

**Published:** 2022-08-19

**Authors:** Benjamin I. Perry, Frederik Vandenberghe, Nathalia Garrido-Torres, Emanuele F. Osimo, Marianna Piras, Javier Vazquez-Bourgon, Rachel Upthegrove, Claire Grosu, Victor Ortiz-Garcia De La Foz, Peter B. Jones, Nermine Laaboub, Miguel Ruiz-Veguilla, Jan Stochl, Celine Dubath, Manuel Canal-Rivero, Pavan Mallikarjun, Aurélie Delacrétaz, Nicolas Ansermot, Emilio Fernandez-Egea, Severine Crettol, Franziska Gamma, Kerstin J. Plessen, Philippe Conus, Golam M. Khandaker, Graham K. Murray, Chin B. Eap, Benedicto Crespo-Facorro

**Affiliations:** a Department of Psychiatry, University of Cambridge, Cambridge, England, United Kingdom; b Cambridgeshire and Peterborough NHS Foundation Trust, Cambridge, England, United Kingdom; c Unit of Pharmacogenetics and Clinical Psychopharmacology, Centre for Psychiatric Neuroscience, Department of Psychiatry, Lausanne University Hospital and University of Lausanne, Prilly, Switzerland; d Virgen del Rocío University Hospital, Network Centre for Biomedical Research in Mental Health (CIBERSAM), Institute of Biomedicine of Seville (IBiS), University of Seville, First-episode Psychosis Research Network of Andalusia (Red PEPSur), Spain; e MRC London Institute of Medical Sciences, Institute of Clinical Sciences, Imperial College, Hammersmith Campus, London, England, United Kingdom; f Department of Psychiatry, Marques de Valdecilla University Hospital, Institute of Biomedicine Marqués de Valdecilla (IDIVAL), Universidad de Cantabria, Santander, Spain; g Institute for Mental Health and Centre for Human Brain Health, University of Birmingham, Birmingham, England, United Kingdom; h Early Intervention Service, Birmingham Womens and Childrens NHS Foundation Trust; i Department of Kinanthropology, Charles University, Prague, Czech Republic; j Les Toises Psychiatry and Psychotherapy Centre, Lausanne, Switzerland; k Service of Child and Adolescent Psychiatry, Department of Psychiatry, Lausanne University Hospital, University of Lausanne, Prilly, Switzerland; l Service of General Psychiatry, Department of Psychiatry, Lausanne University Hospital, University of Lausanne, Prilly, Switzerland; m Centre for Academic Mental Health, Population Health Sciences, Bristol Medical School, University of Bristol, Bristol, England, United Kingdom; n MRC Integrative Epidemiology Unit, Population Health Sciences, Bristol Medical School, University of Bristol, Bristol, England, United Kingdom; o School of Pharmaceutical Sciences, University of Geneva, Geneva, Switzerland; p Center for Research and Innovation in Clinical Pharmaceutical Sciences, Lausanne University Hospital and University of Lausanne, Switzerland; q Institute of Pharmaceutical Sciences of Western Switzerland, University of Geneva, University of Lausanne, Geneva, Switzerland

**Keywords:** Psychosis, Early Intervention, Risk Prediction Algorithm, Metabolic Syndrome, International Validation, PsyMetab, PAFIP

## Abstract

**Background:**

Cardiometabolic dysfunction is common in young people with psychosis. Recently, the Psychosis Metabolic Risk Calculator (PsyMetRiC) was developed and externally validated in the UK, predicting up-to six-year risk of metabolic syndrome (MetS) from routinely collected data. The full-model includes age, sex, ethnicity, body-mass index, smoking status, prescription of metabolically-active antipsychotic medication, high-density lipoprotein, and triglyceride concentrations; the partial-model excludes biochemical predictors.

**Methods:**

To move toward a future internationally-useful tool, we externally validated PsyMetRiC in two independent European samples. We used data from the PsyMetab (Lausanne, Switzerland) and PAFIP (Cantabria, Spain) cohorts, including participants aged 16–35y without MetS at baseline who had 1–6y follow-up. Predictive performance was assessed primarily via discrimination (C-statistic), calibration (calibration plots), and decision curve analysis. Site-specific recalibration was considered.

**Findings:**

We included 1024 participants (PsyMetab *n=*558, male=62%, outcome prevalence=19%, mean follow-up=2.48y; PAFIP *n*=466, male=65%, outcome prevalence=14%, mean follow-up=2.59y). Discrimination was better in the full- compared with partial-model (PsyMetab=full-model C=0.73, 95% C.I., 0.68–0.79, partial-model C=0.68, 95% C.I., 0.62–0.74; PAFIP=full-model C=0.72, 95% C.I., 0.66–0.78; partial-model C=0.66, 95% C.I., 0.60–0.71). As expected, calibration plots revealed varying degrees of miscalibration, which recovered following site-specific recalibration. PsyMetRiC showed net benefit in both new cohorts, more so after recalibration.

**Interpretation:**

The study provides evidence of PsyMetRiC's generalizability in Western Europe, although further local and international validation studies are required. In future, PsyMetRiC could help clinicians internationally to identify young people with psychosis who are at higher cardiometabolic risk, so interventions can be directed effectively to reduce long-term morbidity and mortality.

**Funding:**

NIHR Cambridge Biomedical Research Centre (BRC-1215-20014); The Wellcome Trust (201486/Z/16/Z); Swiss National Research Foundation (320030-120686, 324730- 144064, and 320030-173211); The Carlos III Health Institute (CM20/00015, FIS00/3095, PI020499, PI050427, and PI060507); IDIVAL (INT/A21/10 and INT/A20/04); The Andalusian Regional Government (A1-0055-2020 and A1-0005-2021); SENY Fundacion Research (2005-0308007); Fundacion Marques de Valdecilla (A/02/07, API07/011); Ministry of Economy and Competitiveness and the European Fund for Regional Development (SAF2016-76046-R and SAF2013-46292-R).

For the Spanish and French translation of the abstract see Supplementary Materials section.

**Research in context**

**Evidence before this study**

Meta-analyses have consistently found strong global associations between cardiometabolic and psychotic disorders. This directly translates to a global shortened life-expectancy of up to 15 years in people with psychotic disorders. In the general population, cardiometabolic risk prediction algorithms are commonly used to encourage personalized treatment decisions with the aim of primary prevention of longer-term cardiometabolic outcomes. However, a recent systematic review and exploratory analysis found that these existing algorithms are unlikely to be suitable for young people with psychosis and may underpredict risk in this group. Therefore, a cardiometabolic risk prediction algorithm tailored for young people with psychosis, the Psychosis Metabolic Risk Calculator (PsyMetRiC) was developed and externally validated in the UK.

**Added value of this study**

Risk prediction algorithms can only be confirmed to be suitable for populations they have been tested in, and international populations are likely to vary in ethnicity; culture and dietary habits; average population health, healthcare access; social norms, behaviours and attitudes, and legislation. We therefore performed detailed external validation analysis of PsyMetRiC in two independent European samples, from Switzerland and Spain. In doing so, we found that PsyMetRiC maintains its predictive performance and potential clinical usefulness across those borders and can reliably predict the risk of incident metabolic syndrome in young people with psychosis.

**Implications of all the available evidence**

PsyMetRiC is likely to be generalizable for use in at least some Western European nations. Our findings can pave the way toward a future globally-useful bedside tool to encourage personalized treatment decisions with the aim of improving the long-term physical health of young people with psychosis.

Alt-text: Unlabelled box

## Background

People with psychotic disorders such as schizophrenia die on average 10–15 years sooner than the general population,^1^ predominantly due to a substantial burden of physical comorbidity including type 2 diabetes (T2D), obesity and cardiovascular disease (CVD).^2^ Crucially, this comorbidity reduces quality of life, is responsible for a considerable proportion of overall treatment costs,^3^ and it transcends borders: a high prevalence of cardiometabolic disorders has been consistently reported among people with psychotic disorders in Europe,^4^, ^5^, ^6^, ^7^ The Americas,^8^^,^^9^ Oceania,^10^ Asia^11^ and Africa.^12^

An early marker of cardiometabolic risk is the metabolic syndrome (MetS), which is a clustering of cardiometabolic traits such as disrupted glucose-insulin homeostasis, adiposity, hypertension, and dyslipidaemia. While MetS is a cardiometabolic intermediate, it has consistently shown a high risk of progression to more distal and chronic phenotypes such as type 2 diabetes^13^ and cardiovascular disease,^14^ alongside severe disease endpoints such as myocardial infarction,^15^ cerebrovascular events^16^ and premature mortality.^17^ Therefore, the metabolic syndrome is an important marker of past, present, and future cardiometabolic risk. Treatment for MetS usually focuses on addressing the relevant constituent traits either through behavioural or pharmacological interventions.

Large-scale meta-analyses confirm a globally high prevalence of MetS in young people with psychosis,^18^ and those trends translate into strong global associations with CVD.^19^ Therefore, there is an international need for new strategies to address the physical comorbidity of psychotic disorders.

The comorbidity between psychotic and cardiometabolic disorders begins early. Disrupted glucose-insulin homeostasis may pre-date the onset of psychosis,^20^ and clinically-relevant insulin resistance and dyslipidaemia are detectable from the onset of psychosis in relatively young antipsychotic naïve patients.^21^^,^^22^ Since pharmacological treatments for psychotic disorders can further exacerbate cardiometabolic dysfunction,^23^ it is crucial that young patients who are most at risk of adverse cardiometabolic outcomes are identified at the outset, so interventions can be directed in an informed manner. Yet, a recent systematic review of cardiometabolic risk prediction algorithms developed either for the general or psychiatric populations reported that none were likely to be suitable for young people with psychotic disorders, and commonly used algorithms substantially underpredict risk in this group.^24^

Recently, the first cardiometabolic risk prediction algorithm specifically tailored for young people with psychotic disorders, the Psychosis Metabolic Risk Calculator (PsyMetRiC), was developed in the UK.^25^ When externally validated in another UK sample, PsyMetRiC reliably predicted up-to six-year risk of metabolic syndrome, an age-appropriate precursor to CVD and early mortality.^26^ PsyMetRiC was designed to be clinically-useful and acceptable to young people, using only commonly recorded data. PsyMetRiC consists of two versions, the full- and partial-models, with the latter excluding biochemical predictors to cover situations where blood tests results are not available. PsyMetRiC demonstrated greater net-benefit than competing strategies across a range of feasible risk-thresholds,^25^ suggesting it may be clinically useful and aid in the potential to intervene before MetS has developed, reducing the chance of it developing at all.

However, prognostic algorithms can only be confirmed to be suitable for populations they have been tested in. International populations are likely to vary in ethnicity; culture and dietary habits; average population health, healthcare access; social norms, behaviours and attitudes, and legislation. Even large-scale general population-based cardiometabolic risk prediction algorithms show varying performance when tested in different populations.^27^, ^28^, ^29^

Therefore, following TRIPOD reporting guidelines^30^ (Supplementary Data), we conducted the first study to examine the international transportability of PsyMetRiC. We explored predictive performance in two independent European samples; considered recalibration approaches to create locally-calibrated PsyMetRiC versions; and examined clinical usefulness. Finally, we examined whether prior antipsychotic exposure impacted the predictive performance of PsyMetRiC.

## Methods

### Data sources

#### PsyMetab (Switzerland)

We used data from PsyMetab^4^: an observational prospective study of psychiatric in- and outpatients ongoing since 2007 in the Department of Psychiatry, Lausanne University Hospital, Switzerland and in a private mental health care centre (Les Toises; Lausanne, Switzerland), approved by the Ethics Committee of the Canton of Vaud. Patients were included in both inpatients and outpatient centres located in a region of about 300,000 inhabitants (Lausanne and the surrounding region). Because recruitment was conducted in different centres and institutions, selection bias in the PsyMetab cohort is low.

Briefly, metabolic parameters were collected alongside sociodemographic and lifestyle information at baseline and at 1, 3 and 12 months, and then yearly after the introduction of psychotropic medication. Informed consent was obtained for all participants, which allowed extraction of data collected before 07/08/2021. In addition, the Ethics Committee of the Canton of Vaud granted access to data of participants in the Department of Psychiatry, Lausanne University Hospital collected before 01/01/2016. The total study population featured 2,852 participants. All analyses of PsyMetab data presented herein were conducted locally in Switzerland using R version 4.1.1.^31^

#### PAFIP (Spain)

We used data from the Programa Asistencial Fases Iniciales de Psicosis (PAFIP) study,^32^ an ongoing longitudinal intervention program of first episode non-affective psychosis patients from “Marqués de Valdecilla” University Hospital, Santander, Spain^33^ since 2001, as approved by the Clinical Research Ethics Committee of Cantabria, Spain. PAFIP is an intensive early intervention service (EIS) aimed at early detection and treatment of first episode non-affective psychosis patients in Cantabria, Northern Spain. Participants or their families provided written informed consent. As a clinical program, PAFIP includes inpatient and outpatient care, and provides specific and personalized clinical attention, cognitive behavioural psychotherapeutic interventions, psychopharmacological treatment for patients, and family interventions during the first 3 years after the program intake.^32^ The total study population featured 885 participants. All analyses of PAFIP data presented herein were conducted locally in Spain using R version 4.1.2.^31^

#### Comparisons between study populations

The UK, Switzerland and Spain are three high-income European countries, and each are highly developed social market economies. While there are similarities between the three countries, there are notable differences also. See Supplementary Methods and Supplementary Table 1 for a detailed comparison of key sociodemographic, economic, and healthcare-related metrics between the overall British, Spanish, and Swiss populations.

Regarding the cohort regions specifically, Cantabria has a population of 535,131 inhabitants, of whom 180,717 live in the capital city, Santander. Of the employed population, 60% work in services, 32% in industry, and 8% in the primary sector. These data are similar to the rest of Spain.^34^ Likewise, unemployment rate trends in Cantabria over the last 40 years is representative of Spain in general, and the gross domestic product per capita in Cantabria ($25,180) is similar to wider Spain ($30,090). Although immigration rates are rising across Spain, Spanish natives still make up around 90% of the population. In Cantabria, it is estimated that around 95% of the population is White European, yet the ethnic makeup of PAFIP is similar to other Spanish psychosis cohorts.^35^ The sex and age distribution in PAFIP cohort is similar to that reported in other Spanish cohorts.^36^ No national study of the incidence of psychosis has been conducted to date in Spain,^37^ but the global annual incidence for psychosis in Cantabria is 1·38/10,000 person years.^33^ This is similar to the median values reported in other Spanish cities.^36^

The Lausanne region of Switzerland has a population of around 300,000, with around 140,000 living in the city itself. Of the employed population, 79% work in services, 19% in industry, and 2% in the primary sector. Migrants make up 30% of the population of the Lausanne region, slightly lower than the national prevalence (38%). The incidence of psychosis in the Lausanne region has not been reported, but the incidence of psychosis across Switzerland is in line with other developed nations.^38^^,^^39^ Cardiometabolic parameters of PsyMetab have been compared to the CoLaus general population cohort, finding a similar prevalence of MetS and obesity.^4^ In addition, national statistics from the Swiss Federal Statistical Office suggest that the prevalence of obesity in PsyMetab is similar to the Swiss general population.^40^ Furthermore, cardiovascular risk in PsyMetab as estimated using the Framingham Risk Score is in line with other psychiatric populations.^4^ Finally, the median socio-economic status in PsyMetab^41^ is similar to the general Swiss population,^42^ suggesting that the cohort is representative of the Swiss population.

### Inclusion and exclusion criteria

Following the methodology from the original PsyMetRiC study,^25^ in both samples we excluded participants who: were aged <16y or >35y at the time of baseline assessment; had <1 year follow-up data available; met the outcome criteria at baseline; or had missing data on all predictor or outcome constituent variables. We also excluded participants who did not have a diagnosis of a psychosis-spectrum disorder at baseline (ICD-10 codes F06·0-2, F20-F31, F32·3, F33·3, F53·1 as defined in the original PsyMetRiC study^25^). See Supplementary Table 2 for the diagnostic classification of included participants. See Supplementary Figure 1 for a flow-chart of included participants in the study from both samples.

### Outcome

As per the original PsyMetRiC study,^25^ we used the harmonized definition^43^ of MetS as a binary outcome: ethnicity-specific waist circumference ≥94 cm in males and ≥80 cm in females for Caucasians; ≥90 cm in males and ≥80 cm in females for other ethnic groups, or body mass index (BMI) >29.9; alongside two of: triglycerides ≥1.70mmol/L; high-density lipoprotein (HDL) <1.03mmol/L (males) or <1.29mmol/L (females); systolic blood pressure >130 mmHg; fasting plasma glucose (FPG) >5.60mmol/L. In each sample, where multiple follow-ups were available for each participant, we used the latest follow-up available between 1 and 6 years after baseline with the least amount of missing data (Statistical Analysis).

### The PsyMetRiC algorithms

PsyMetRiC consists of two forced-entry multivariable penalized logistic regression equations: the full-model and the partial-model. Predictors were included on a balance of clinical knowledge, prior research, and likely clinical usefulness/patient acceptability. See the original PsyMetRiC study^25^ for further details. The partial-model was developed to cover eventualities where biochemical results may not be available. The PsyMetRiC algorithm coefficients are presented in Table 1. See the original PsyMetRiC study^25^ for further details. See Supplementary Table 3 for the associations of individual PsyMetRiC predictors with MetS in the PsyMetab and PAFIP samples.

**Table 1:** Original PsyMetRiC Algorithm Coefficients After Shrinkage for Optimism.

PsyMetRiC Predictor	Full-Model	Partial-Model
Intercept	−6.439813	−6.973829
Age in years (continuous)	0.006233226	0.00633115
Black/African-Caribbean Ethnicity (yes/no)	0.004258861	0.07548129
Asian / Other Ethnicity (yes/no)	0.211217746	0.29285950
Male Sex (yes/no)	0.222300765	0.31460036
Body Mass Index (BMI) (kg/m^2^) (continuous)	0.141186241	0.16912161
Current Smoking Status (smoker, non-smoker)	0.153691193	0.24751854
Prescribed a Metabolically-Active Antipsychotic^a^ (yes/no)	0.497552758	0.60013558
High-Density Lipoprotein (HDL) (mmol/L) (continuous)	−0.399013329	b
Triglycerides (mmol/L) (continuous)	0.343528440	b

a See Supplementary Table 4.

b Predictor not included in model.

### Statistical analysis

#### Sample preparation and estimation of analytic precision

Biochemical values were converted to mmol/L where necessary. We assessed for the presence of predictor multi-collinearity in both samples by measuring the variance inflation factor (Supplementary Methods). Recently developed criteria^44^ to estimate analytic precision given the fixed sample sizes (Supplementary Methods) were applied. Briefly, the expected SEs for the C-statistic were 0.028 (PsyMetab) and 0.029 (PAFIP). The expected SEs for the calibration slope and calibration-in-the-large were 0.14 & 0.13 (PsyMetab), and 0.15 & 0.13 (PAFIP) respectively. Multiple imputation using chained equations was considered for missing data (Supplementary Methods). For numerical-based analyses, estimates were pooled using Rubin's rules. For plot-based analyses, plots were generated in each imputed dataset and checked for similarity, with one randomly selected plot per analysis presented in the main manuscript and all remaining plots presented in the Supplementary Data. Comparisons between the original PsyMetRiC development sample, PsyMetab and PAFIP samples for key sociodemographic, lifestyle and biochemical characteristics were performed using ANOVA (for means) and the chi-square equality of proportions test (for proportions).

#### Primary external validation analysis

The algorithms were applied to both samples independently. The distribution of predicted outcome probabilities was inspected using histograms. Algorithm performance was primarily assessed with measures of discrimination (concordance (C-) statistic), and calibration (calibration plots) (Supplementary Methods). We also recorded the Nagelkerke-Cox-Snell-Maddala-Magee r^2^ index, the calibration intercept (ideally close to 0), calibration slope (ideally close to 1), and the Brier score (ideally close to 0, with scores >0.25 indicating poor performance).

#### Recalibration and generation of site-specific PsyMetRiC versions

Given the challenges of external validation in international samples, we expected differences in calibration performance compared with the original PsyMetRiC study, which was developed in the UK. We considered a logistic calibration approach in instances where we identified miscalibration (i.e., unfavourable agreement between the observed proportion and predicted probability) on visual inspection of calibration plots. Logistic calibration takes into account differences in baseline risk that may exist between populations by re-estimating the intercept term, and also re-estimates the slope term. Therefore, logistic calibration assumes similar relative effects of the predictors but allows for larger or smaller absolute effects of the predictors.^45^ By completing this step, we obtained site-specific versions of PsyMetRiC (PsyMetRiC-CH and PsyMetRiC-ES) (Supplementary Methods). For all results in our analysis, as is customary in prediction modelling research, we present performance estimates accompanied by 95% CIs (derived from an alpha-value of 0.05 as commonly used in inferential statistics). However, in instances where recalibration of PsyMetRiC was conducted, as an additional sensitivity analysis we also present estimates accompanied by an adjusted confidence interval threshold. For example, where analysis was performed before and after logistic recalibration, we divided the “alpha-level” by 2 (0.05/2=0.025) and so present estimates alongside 99% confidence intervals.

#### Clinical usefulness

Decision curve analysis^46^ was used to assess clinical usefulness by estimating net benefit across a range of feasible thresholds (i.e., the risk score at which an intervention would be deemed necessary) (Supplementary Methods). We considered a risk threshold upper-bound of 0.30, which represents around a one-in-three chance of developing MetS should nothing change, because it is unlikely that risk thresholds greater than that would be tolerated without intervention. Net benefit incorporates the consequences of the decisions made on the basis of an algorithm, and is therefore preferable to related measures such as sensitivity and specificity.^47^ We reported the net benefit and standardized net benefit (net benefit / outcome prevalence, i.e., the additional percentage of cases that could be intervened on with use of PsyMetRiC with no increase in false-positives) across a range of reasonable risk thresholds. We drew a decision curve plot to visualise and compare the net benefit of the original vs the site-specific PsyMetRiC versions in each sample, compared with intervening in all or intervening in none. Classical decision theory proposes that at a chosen risk-threshold, the choice with the greatest net-benefit should be preferred.^47^

#### Sensitivity analyses

We performed a missing sample comparison to assess the potential impact of missing data on our results. We also examined whether nil or previous use of antipsychotic medications may affect predictive performance by repeating the analysis after excluding participants who were not antipsychotic naïve at the baseline assessment.

#### Data visualisation

An online data visualisation website for PsyMetRiC was created to accompany the original study (https://psymetric.shinyapps.io/psymetric). The website was updated with site-specific PsyMetRiC versions obtained through recalibration analysis. We also prepared two simulated case scenarios presented as decision trees to visualise the impact of modifiable and non-modifiable risk factors in young people with psychosis, as calculated from the recalibrated site-specific PsyMetRiC full- and partial-models (Supplementary Figure 10).

### Role of the funding sources

The funding sources had no role in the study design, data collection, analysis, interpretation or writing of the manuscript.

## Results

### Samples

After applying inclusion criteria, we included *n*=558 from the PsyMetab cohort, and *n*=466 from the PAFIP cohort (Table 2). The PsyMetab and PAFIP samples differed from each other and from the original UK PsyMetRiC development sample on most sociodemographic, lifestyle and biochemical characteristics (Table 2).

**Table 2:** Sociodemographic characteristics of the original psymetric development sample and included external validation samples.

Characteristic	Original PsyMetRiC Development Sample (UK)	PsyMetab External Validation Sample (Switzerland)	PAFIP External Validation Sample (Spain)	Between-Group Differences^d^
Sample before Inclusion/Exclusion Criteria Applied^a^, *N.*	1504	2852	885	-
Included sample size^a^, *N.* (%)	651 (43.28)	558 (19.57)	466 (52.66)	-
Age in Years, mean (SD)	24.52 (4.91)	25·92 (5.32)	25·51 (4.99)	F=12.22, *p*<0.0001
White European/NR Ethnicity, *N.* (%)	360 (55.3)	446 (79.93)	435 (93.34)	χ=219.67, *p*<0.0001
Black/African-Caribbean Ethnicity, *N.* (%)	109 (16.74)	68 (12.19)	15 (3.22)	χ=49.38, *p*<0.0001
Asian/Other Ethnicity, *N.* (%)	181 (27.80)	44 (7.48)	16 (3.43)	χ=159.67, *p*<0.0001
Male Sex, *N.* (%)	440 (67.59)	345 (61.83)	303 (65.16)	χ=4.38, *p=*0.112
HDL at baseline, mmol/L, mean (SD)	1.88 (0.57)	1.33 (0.36)	1.32 (0.34)	F=303.57, *p*<0.0001
Triglycerides at baseline, mmol/L, mean (SD)	1.39 (1.06)	1.16 (0.70)	0.88 (0.40)	F=54.89, *p*<0.0001
BMI at baseline, kg/m^2^, mean (SD)	23.63 (5.43)	23.60 (5.00)	22.50 (3.36)	F=9.14, *p*<0.0001
FPG at baseline (mmol/L), mean (SD)	5.19 (1.28)	4.95 (0.82)	4.69 (0.55)	F=36.11, *p*<0.0001
Systolic BP at baseline (mmHg), mean (SD)	120.65 (11.68)	121.32 (14.00)	119.86 (14.10)	F=1.56, *p*=0.211
Prescribed a More-Metabolically-Active Antipsychotic^b^, *N.* (%)	455 (69.89)	413 (74.01)	234 (50.21)	χ=71.87, *p*<0.0001
Smoking at baseline, *N*. (%)	315 (48.39)	362 (64.87)	279 (59.90)	χ=35.40, *p*<0.0001
Follow-up time, years, mean (SD)	1.86 (1.32)	2.48 (1.40)	2.59 (0.73)	F=61.47, *p*<0.0001
Antipsychotic Naïve at baseline, *N*. (%)	NR	361 (64.70)	433 (92.92)	χ=114.53, *p*<0.0001
Metabolic Syndrome at baseline, *N*. (%)^c^	49 (6.58)	36 (6.06)	31 (6.24)	χ=0.53, *p*=0.766
Metabolic Syndrome at Follow-up, *N*. (%)	109 (16.74)	103 (18.54)	66 (14.16)	χ=3.40, *p*=0.183

HDL=high-density lipoprotein; BMI=body mass index; FPG=fasting plasma glucose; BP=blood pressure; NR=Not recorded; d.f.=degrees of freedom.

a See Supplementary Figure 1 for a flow-chart of included participants in the study.

b Definitions of Metabolically-active antipsychotics are listed in Supplementary Table 1.

c Corresponds to percentage of sample before those participants were excluded.

d Analysis of means was conducted using one-way ANOVA. Analysis of proportions was conducted using the chi-square equality of proportions test.

### Primary external validation analysis

#### PsyMetab, Switzerland

**Figure 1:**
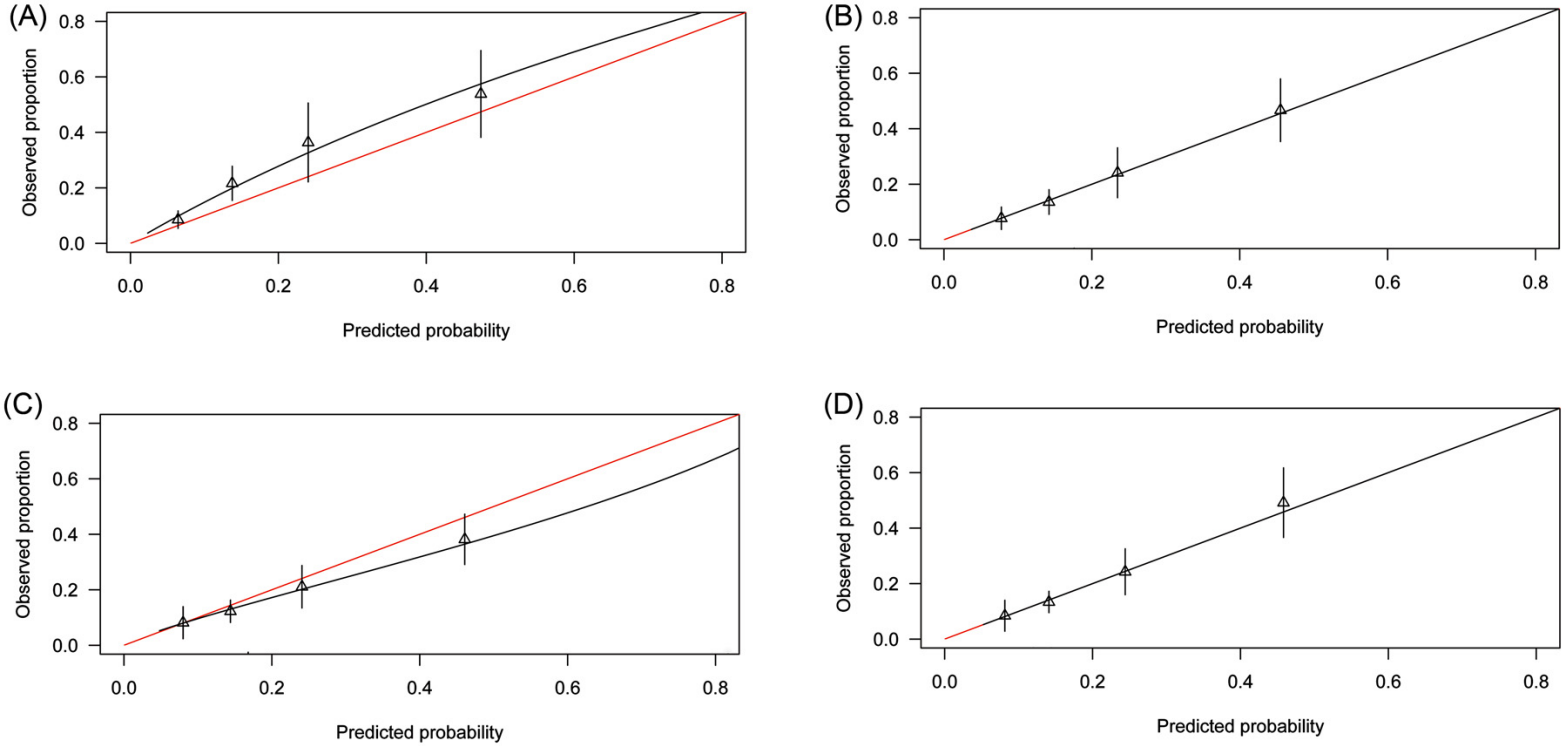
**Calibration Plots of PsyMetRiC in PsyMetab (Switzerland)**. A = Primary Analysis - Full Model; B = After Logistic Calibration – Full Model; C = Primary Analysis – Partial Model; D = After Logistic Calibration – Partial Model. Calibration plots illustrate agreement between the observed (y axis) and predicted risk (x axis). Perfect agreement would trace the red line. Algorithm calibration is illustrated by the black line. Triangles denote grouped observations for participants at deciles of predicted risk, with 95% C.I.’s indicated by the vertical black lines. ^a^Logistic calibration takes into account differences in baseline risk that may exist between populations by re-estimating the intercept term, and also re-estimates the slope term thus assuming similar *relative* effects of the predictors but allowing for a larger or smaller *absolute* effect of the predictors. See Methods. (For interpretation of the references to color in this figure legend, the reader is referred to the web version of this article.)

The shape of the distribution of predicted probabilities was similar to the original PsyMetRiC study (Supplementary Figure 2). Predictive performance statistics are reported in Table 3. Calibration plots for the full-model were similar across imputed datasets (Figure 1; Supplementary Figure 3) and show a systematic minor degree of risk underprediction. For the partial-model, calibration plots were similar across imputed datasets and show a minor degree of risk overprediction at higher predicted probabilities (Figure 1; Supplementary Figure 3).

**Table 3:** Predictive performance statistics of the PsyMetRiC full- and partial models before and after logistic calibration in PsyMetab and PAFIP.

Measure of Predictive Performance	Primary Analysis, Estimate (95% C.I.)		After Logistic Calibration, Estimate (95% C.I.)	
	Full-Model	Partial-Model	Full-Model	Partial-Model
**PsyMetab (Switzerland)**				
C-Statistic	0.73 (0.68, 0.79)	0.68 (0.62, 0.74)	0.73 (0.68, 0.79)	0.68 (0.62, 0.74)
r^2^	0.10 (0.06, 0.14)	0.08 (0.03, 0.13)	0.12 (0.09, 0,15)	0.08 (0.04, 0.12)
Calibration Intercept	0.11 (−0.05, 0.26)	0.12 (0.05, 0.19)	−0.01 (−0.01, −0.01)	−0.01 (−0.01, −0.01)
Calibration Slope	0.77 (0.72, 0.82)	0.93 (0.86, 1.00)	1.02 (1.01, 1.04)	1.03 (1.01, 1.05)
Brier Score	0.13 (0.09, 0.17)	0.14 (0.08, 0.20)	0.13 (0.08, 0.16)	0.14 (0.10, 0.18)
**PAFIP (Spain)**				
C-Statistic	0.72 (0.66, 0.78)	0.66 (0.60, 0.71)	0.72 (0.66, 0.78)	0.66 (0.60, 0.71)
r^2^	0.10 (0.05, 0.15)	0.05 (0.02, 0.08)	0.10 (0.05, 0.15)	0.05 (0.02, 0.08)
Calibration Intercept	0.24 (0.09, 0.38)	−0.30 (−0.38, −0.22)	0.01 (0.00, 0.01)	0.01 (0.00, 0.01)
Calibration Slope	1.09 (0.99, 1.20)	0.91 (0.80, 1.02)	1.03 (1.01, 1.05)	1.04 (1.01, 1.06)
Brier Score	0.13 (0.08, 0.16)	0.12 (0.09, 0.16)	0.11 (0.07, 0.16)	0.12 (0.09, 0.16)

The C-statistic is a measure of discrimination and estimates the probability that a randomly selected ‘case’ will have a higher predicted probability than a randomly selected non-case. Scores of 1.0 indicate perfect discrimination; scores of >0.70 are generally considered acceptable. The calibration intercept (ideally close to 0) and calibration slope (ideally close to 1) are estimates of model calibration (i.e., the agreement between the observed proportion and predicted risk). The Brier score (ideally close to 0, with scores >0.25 indicating poor performance) is an overall measure of algorithm performance. For comparison, results from the original PsyMetRiC external validation in the UK were: full-model: C=0.75 (95% C.I., 0.69–0.80; r2=0.21 (95% CI., 0.18–0.25); Brier score=0.07 (95% C.I., 0.04–0.10); intercept=-0.05 (95% C.I., −0.08, −0.02); partial-model: C=0.74 (95% C.I., 0.67–0.79); r2=0.17 (95% C.I., 0.14–0.20); Brier score=0.08 (95% C.I., 0.05–0.11); intercept=-0.07 (95% C.I., −0.11, −0.03). See the original PsyMetRiC manuscript for further details.^25^

#### PAFIP, Spain

**Figure 2:**
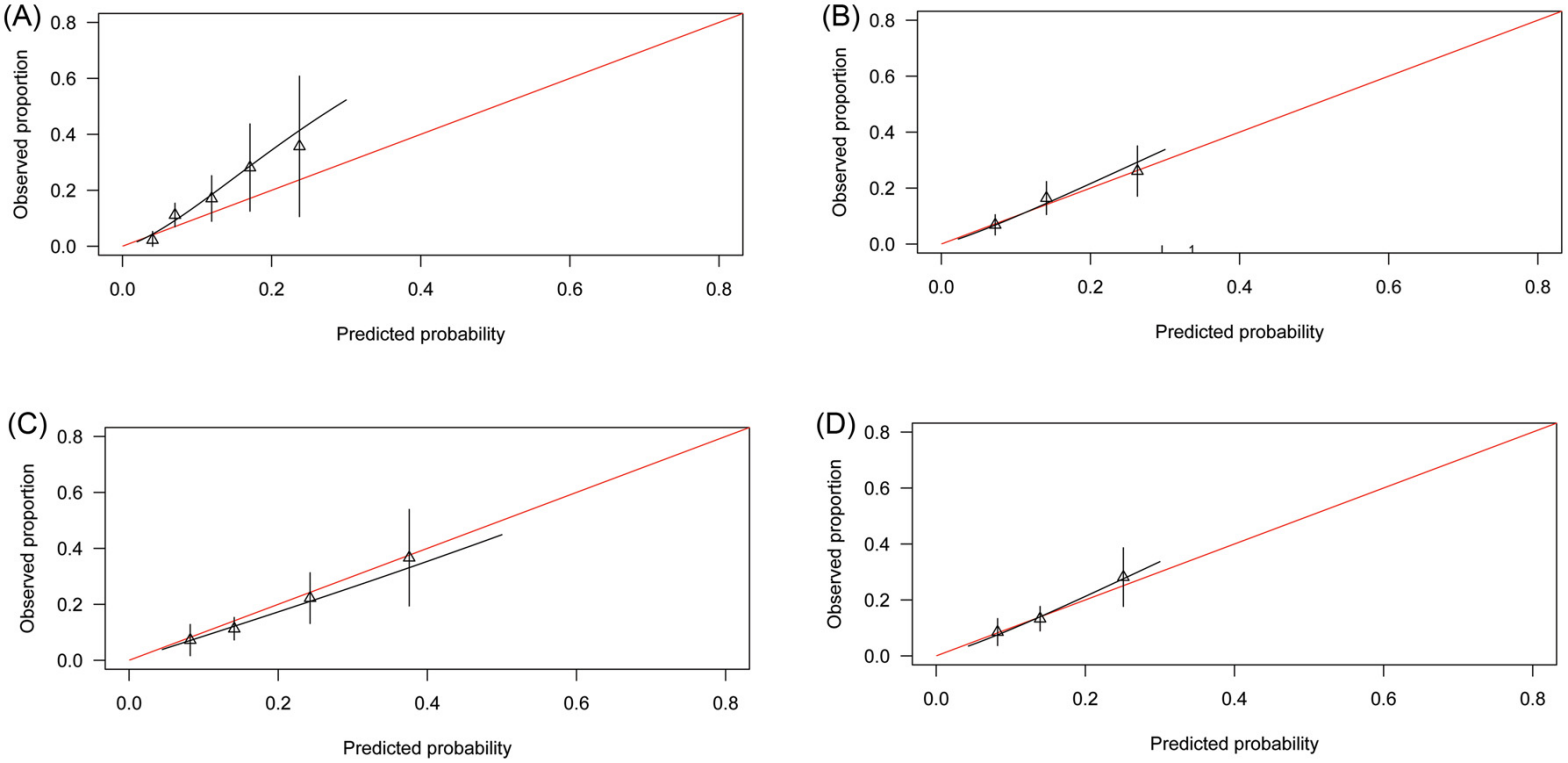
**Calibration Plots of PsyMetRiC in PAFIP (Spain)**. A = Primary Analysis - Full Model; B = After Logistic Calibration – Full Model; C = Primary Analysis – Partial Model; D = After Logistic Calibration – Partial Model. Calibration plots illustrate agreement between the observed (y axis) and predicted risk (x axis). Perfect agreement would trace the red line. Algorithm calibration is illustrated by the black line. Triangles denote grouped observations for participants at deciles of predicted risk, with 95% C.I.’s indicated by the vertical black lines. ^a^Logistic calibration takes into account differences in baseline risk that may exist between populations by re-estimating the intercept term, and also re-estimates the slope term thus assuming similar *relative* effects of the predictors but allowing for a larger or smaller *absolute* effect of the predictors. See Methods. (For interpretation of the references to color in this figure legend, the reader is referred to the web version of this article.)

The shape of the distribution of predicted probabilities was similar to the original PsyMetRiC study (Supplementary Figure 4). Performance statistics are shown in Table 3. Calibration plots for the full-model were similar across imputed datasets (Figure 2; Supplementary Figure 5) and show a systematic degree of marked risk underprediction becoming more severe at higher predicted probabilities. For the partial-model, calibration plots were similar across imputed datasets and show good calibration (Figure 2; Supplementary Figure 5).

### Algorithm recalibration and generation of site-specific PsyMetRiC versions

#### PsyMetab (Switzerland)

After logistic calibration (Supplementary Table 5), the shape of the distributions of predicted probabilities were similar to the primary analysis (Supplementary Figure 2). Recalibrated performance statistics are reported in Table 3 and Supplementary Table 6. Calibration plots for both PsyMetRiC versions were similar across imputed datasets (Figure 1; Supplementary Figure 6) and showed excellent calibration.

#### PAFIP (Spain)

After logistic calibration (Supplementary Table 5), the shape of the distributions of predicted probabilities were similar to the primary analysis (Supplementary Figure 4). Recalibrated performance statistics are shown in Table 3 and Supplementary Table 6. Calibration plots for both PsyMetRiC versions were similar across imputed datasets (Figure 2; Supplementary Figure 7) and showed excellent calibration.

#### Clinical usefulness

**Figure 3:**
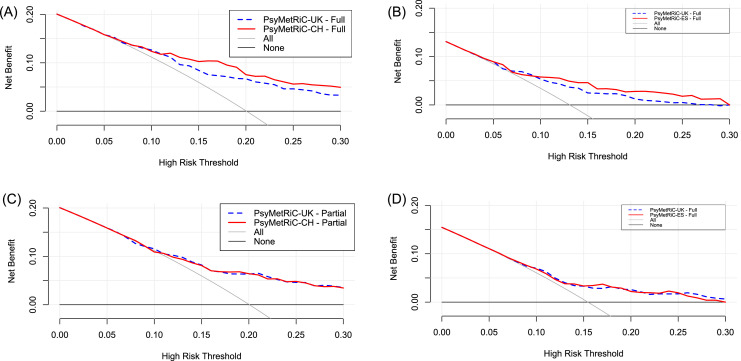
**Clinical Usefulness of PsyMetRiC in The PsyMetab and PAFIP Samples Before and After Logistic Calibration**. A = Full-Model – PsyMetab (Switzerland); B = Full-Model – PAFIP (Spain); C = Partial-Model – PsyMetab (Switzerland); D = Partial-Model – PAFIP (Spain). The plot reports net benefit (y axis) of PsyMetRiC Full- and Partial-Models (blue dotted line = original PsyMetRiC algorithm applied to the sample; red solid line = recalibrated site-specific version) across a range of risk thresholds (x axis) compared with intervening in all (grey solid line) or intervening in none (black solid line). In Decision Curve Analysis, it is customary to consider only the range of risk-thresholds that may reasonably be considered in clinical practice. Our upper bound of 0.30 represents around a one-in-three chance of developing MetS should nothing change, and it is unlikely that risk thresholds greater would be tolerated. Net harm (i.e., more false positives than true positives exposed to an intervention at a selected risk threshold) is indicated when the decision curve line is plotted at y<0. (For interpretation of the references to color in this figure legend, the reader is referred to the web version of this article.)

Decision curve analysis (Figure 3, Supplementary Figure 8) showed that in both samples, PsyMetRiC provided universally greater net benefit than competing strategies. In both samples, net benefit was greater with the full-model compared with the partial model. For example, in PsyMetab, if an intervention was considered for participants scoring higher than 0.15, the recalibrated full- and partial-models provided net benefits of 0.09 (95% C.I., 0.05–0.12) and 0.07 (95% C.I., 0.04–0.10) respectively, meaning that an additional 49% of metabolic syndrome cases could be prevented with the full-model, and 38% with the partial-model. In PAFIP, at the same risk-threshold, the recalibrated full- and partial-models provided net benefits of 0.04 (95% C.I., 0.02–0.07) and 0.03 (95% C.I., 0.01–0.06) respectively, meaning that an additional 30% of metabolic syndrome cases could be prevented with the full-model, and 23% with the partial-model (Supplementary Tables 7–10). Recalibration of PsyMetRiC provided minor improvements to net benefit universally, which was more prominent with the full-model (Figure 3, Supplementary Figure 7, Supplementary Tables 7–10).

### Sensitivity analysis

In both cohorts, participants in the excluded sample were more likely to be older (likely due to the exclusion criteria), female, antipsychotic naïve, more likely to smoke, have a longer follow-up time, and have higher concentrations of HDL, triglycerides and FPG at baseline than participants in the included sample (Supplementary Tables 11–12).

We found that 361 (64.70%) participants of PsyMetab and 433 participants (92.92%) of PAFIP had no recorded prior use of antipsychotics at baseline assessment. The sociodemographic characteristics of those participants were similar compared with the main analysis (Supplementary Tables 13-14), but the prevalence of MetS at follow-up was slightly lower (16.90% vs 18.54% in PsyMetab, 13.16% vs 14.16% in PAFIP). Results for discrimination (PsyMetab: full-model C=0.75, 95% C.I., 0.69–0.82; partial-model: C=0.71, 95% C.I., 0.63–0.78; PAFIP: full-model C=0.72, 95% C.I., 0.65–0.79; partial-model: C=0.66, 95% C.I., 0.65–0.74) and calibration (Supplementary Figure 9) were similar in the antipsychotic naïve subsamples compared with the main analytic samples.

## Discussion

We tested whether the PsyMetRiC cardiometabolic risk prediction algorithm for young people with psychotic disorders, which was developed in the UK, may be generalizable and clinically useful internationally. To do this, we performed detailed external validation analyses in two independent European samples that differed from one another and from the original PsyMetRiC development sample on a range of key sociodemographic, lifestyle and biochemical characteristics. Our results suggest that PsyMetRiC is likely to be generalisable outside of the UK, to at least some Western European nations.

We found that the discrimination performance of PsyMetRiC, as measured by the C-statistic, was similar across both samples, but as expected, slightly reduced compared with the external validation performance in the UK. As in the original PsyMetRiC study,^25^ we also found that the PsyMetRiC full-model discriminated cases of MetS from non-cases better than the partial-model. The better performance of the full-model which included standard blood biochemical results, reiterates that a comprehensive physical health assessment for young people with psychosis should include blood tests where possible. For example, unmedicated patients with first episode psychosis commonly present with metabolic abnormalities including insulin resistance^21^ and dyslipidaemia^22^ even in the presence of a normal BMI.^20^ The partial-model is unable to capture this metabolic abnormality, whereas the full-model is able, because a raised triglyceride:HDL ratio is indicative of insulin resistance^48^^,^^49^ and is clinically useful given that more sensitive tests, such as the homeostasis model assessment or hyperinsulinaemic-euglycaemic clamp method are not routinely available in clinical practice.

The agreement between observed and predicted risk, i.e., calibration, is equally important to algorithm predictive performance. Precise agreement between the observed and predicted risk estimates is crucial since in the future, PsyMetRiC score cut-offs may be introduced to determine eligibility (or ineligibility) for a particular intervention. For older adults in the general population, a QRISK2^50^ score higher than 0·10 defines the need for clinical intervention, which may include prescription of a statin.^51^ The clinical usefulness of QRISK2 at different proposed risk thresholds has been assessed.^52^ An algorithm that is poorly calibrated may therefore lead to the disproportionate withholding of potentially effective interventions in some people who would benefit, and/or *vice versa*, thus potentially predisposing to patient harm. In our study, we found that the calibration performance of both PsyMetRiC versions was good in the PsyMetab cohort, suggesting that the PsyMetRiC risk estimates were likely to be relatively precise in that sample. However, in the PAFIP sample the full-model showed evidence of miscalibration such that PsyMetRiC underpredicted risk. This pattern of miscalibration was not evident with the partial-model, raising the possibility that the biochemical predictors included in the full-model may be one potential explanation for the miscalibration. Interestingly, the mean concentrations of triglycerides were lower in the PAFIP sample compared with the UK development and PsyMetab samples. This between-sample variability may be one contributing explanation for the miscalibration, because the algorithm coefficient for triglycerides was trained and weighted on the UK sample distribution of triglyceride levels. Therefore, the linear predictors derived from the PsyMetRiC equations may have been smaller than expected in the PAFIP sample because the distribution of triglyceride levels were lower than expected, leading to a pattern of underprediction of risk.

Despite this, calibration performance recovered fully following recalibration, without impacting discrimination performance. Nevertheless, even though our recalibration approach can be considered a relatively minor means of algorithm revision because predictor coefficients were unaltered, any change made to a previously validated algorithm necessitates the need for new external validation. Therefore, both site-specific PsyMetRiC versions (PsyMetRiC-CH and PsyMetRiC-ES) now require external validation in unseen samples from Switzerland and Spain respectively, to ensure that the site-specific versions are generalizable to the Swiss and Spanish populations, respectively.

We found that the predictive performance of PsyMetRiC did not differ depending on whether participants were antipsychotic naïve at baseline or not. This is an important finding because it was not possible in the original PsyMetRiC study^25^ to discern whether patients were antipsychotic naïve at baseline. That previous antipsychotic exposure had a negligible effect on algorithm predictive performance increases the likely usefulness of PsyMetRiC for psychosis early intervention services, whose newly enrolled patients might equally be referred from a general/family practitioner (and so may be antipsychotic naïve) or from a psychiatric inpatient unit (and so may have been previously exposed to an antipsychotic).

With decision curve analysis we showed that in both samples, both PsyMetRiC versions are likely to be clinically useful, and could lead to improved detection of future MetS cases in young people with psychotic disorders. In future, these individuals could be considered for targeted intervention strategies with the aim of primary prevention of more distal cardiometabolic outcomes like T2D and CVD, thus reducing long-term morbidity and mortality in this group. Decision curve analysis also showed in both samples that the full-model improved net benefit to a greater extent than the partial-model, and also that the recalibrated site-specific versions improved net benefit even further with the full-model. Yet, given that the site-specific versions require additional external validation, our results suggest that the original PsyMetRiC algorithms could be used unamended and still be clinically useful.

Despite the encouraging findings, PsyMetRiC now requires revision to further improve its accuracy, alongside further testing in UK and international samples. Regarding revision, there are several aspects of PsyMetRiC that can be improved. First, the severity of the metabolic adverse effects of different antipsychotics exists as a continuum rather than a dichotomy.^23^ A future revision of PsyMetRiC should seek to include antipsychotics modelled individually. Relatedly, each algorithm predictor is determined at baseline and so the potential impact of future antipsychotic switching due to adverse effects (which may or may not be cardiometabolic) and/or poor efficacy cannot be known. While our defined population consists of individuals early in the course of a psychotic disorder and the mean follow-up time of 2–3 years reduces the likelihood of multiple switches, more complex modelling strategies in sufficiently powered samples will be required to fully address this issue.

A second aspect of the PsyMetRiC algorithm that could be improved is the granularity of the ethnicity predictor. At present due to sample size limitations ethnicity is captured in three categories, yet this is likely to be an oversimplification. For example, there are clear differences in cardiometabolic risk among different Asian ethnic groups,^53^ and between Black Africans and Black Caribbeans.^54^ PsyMetRiC cannot at present capture those differences. Due to sample size limitations, we could not consider examining predictive performance stratified by ethnic group, yet even large-scale general population-based algorithms like QRISK2 and the Framingham score perform worse in ethnic minority groups.^55^ A future refinement of PsyMetRiC must therefore aim to capture ethnicity in greater detail, and this could be achieved in a larger, more ethnically diverse sample which may also permit analysis stratified by ethnicity, to ensure that PsyMetRiC performs equally well for all patients. Furthermore, the prevalence of cardiometabolic disorders differs between native ethnic minority groups compared with migrants,^56^ suggesting other factors are also relevant, such as deprivation. A deprivation score, such as the UK's Townsend deprivation index^57^ might also be considered in future, though this would limit international transportability.

The prevailing strength of this study is the inclusion of two relatively large independent samples from distinct European nations, each well-characterized, permitting detailed external validation analyses. Analyses were conducted independently by researchers locally in Switzerland and Spain. That the results were similar across both samples, and similar to the original PsyMetRiC study fosters confidence in the international transportability of the algorithm. We performed detailed sample size calculations to determine the likely precision of our analyses. We followed best-practice methods^58^ in the conduct and reporting of the analysis and adhered to TRIPOD reporting guidelines.^30^ Nevertheless, the results should be considered together with the following limitations. The PsyMetab study was not designed to include drug-naïve and/or early psychosis patients specifically. Drug-naïve patients were assumed as so based on having no health record of prior antipsychotic exposure. Because other institutions/private practitioners can also introduce these treatments some patients may have been misclassified. Patients with affective psychosis were not included in the PAFIP cohort, though in reality an EIS might not exclude those patients. Both the PsyMetab and PAFIP samples were less ethnically diverse that the original PsyMetRiC study. Selection bias may have affected our analysis, since we excluded participants who had complete missing data at baseline and/or follow-up. This methodological step was deemed preferable to imputing complete participant data. Multiple imputation can be biased when data are missing not at random, although we included auxiliary variables to reduce the fraction of missing information, limiting the effect of this bias. While the sample sizes of PsyMetab and PAFIP were large enough to conduct relatively precise external validation analyses, larger samples in the future will permit analyses stratified by certain protected characteristics, to ensure that the algorithm performs equally well for marginalized or under-represented groups. Finally, PsyMetRiC has only been tested in Western populations so far. To advance toward a truly globally useful tool, PsyMetRiC requires validation in diverse populations.

To conclude, we have performed the first international external validation study of the PsyMetRiC cardiometabolic risk prediction algorithm tailored for young people with psychosis, and the results suggest that its accuracy is likely to be generalizable to at least some Western European nations. This is encouraging, but further validation studies both in the UK and internationally are required before PsyMetRiC can be rolled out for routine use globally.

## Data access statement

Analyses of data from PsyMetab and PAFIP were run independently by researchers in Switzerland and Spain respectively. FV and CBE directly accessed and verified the underlying data for analyses of PsyMetab. NG, VO, JV-B and BC-F directly accessed and verified the underlying data for analyses of PAFIP.

## Contributors

BIP, FV, NG-T: conceptualization, investigation, methodology, project administration, resources, formal analysis, data interpretation, software, visualization, writing - original draft, writing - review & editing; EFO: resources, methodology, writing - review & editing; MP: project administration, data curation, resources, writing – review & editing; JV-B: conceptualization, investigation, project administration, resources, writing - review & editing; RU: conceptualization, supervision, writing - review & editing; CG: project administration, data curation, resources, writing – review & editing; VO: investigation, methodology, resources, formal analysis, visualization, writing - review & editing; PBJ: conceptualization, supervision, writing - review & editing; NL: project administration, data curation, resources, writing – review & editing; MR-V: investigation, methodology, writing - review & editing; JS: methodology, supervision, writing - review & editing; CD: project administration, data curation, resources, writing – review & editing; MC-R: investigation, review - writing & editing; PKM: supervision, writing - review & editing; AR-D: project administration, data curation, resources, writing – review & editing; NA: resources, writing – review & editing; EF-E: supervision, writing - review & editing; SC: resources, writing – review & editing; FG: project administration, resources, writing – review & editing; KVP: project administration, resources, writing – review & editing; PC: project administration, resources, writing – review & editing; GMK, GKM, CBE, BC-F: conceptualization, resources, project administration, supervision, writing - review & editing.

## Data sharing statement

Data from PAFIP and PsyMetab cannot be publicly deposited due to patient and participant confidentiality purposes. Data from PAFIP or PsyMetab can be accessed after formal application to and ethical review by the respective scientific committee. See study group websites for further details (https://psynal.eu/ and http://www.chuv.ch/cnp-psymetab).

## Declaration of interests

RU has in the past 3 years received honoraria for speaking events from Oktuska, Synovion and Vyalife; has participated as a Chair TSC for an NIHR-funded clinical trial on antipsychotic medication for treatment resistant depression, and as an Expert Member for an NIHR-funded clinical trial on psychological therapies for common mental disorders; is honorary general secretary for the British Association of Psychopharmacology and a Deputy Editor for British Journal of Psychiatry. PBJ is Chair of the MQ Mental Health Sciences Council and has participated in an advisory board for MSD on an unrelated mental health topic. FV received in the past 3 years honoraria for conferences or teaching CME courses from Forum für MedizinischeFortbildung and Sysmex Suisse AG. NA received in the past 3 years honoraria for a conference from Sysmex Suisse AG. SC received in the past 3 years honoraria for teaching CME courses from Forum pour la formation médicale, Barr Switzerland and for consultancy from the Swiss Health Observatory (Obsan) of the Swiss Federal Office of Public Health. CBE received in the past 3 years honoraria for conferences or teaching CME courses from Janssen-Cilag, Lundbeck, Otsuka, Sandoz, Servier, Sunovion, Sysmex Suisse AG, Takeda, Vifor-Pharma and Zeller. All other authors declare no potential conflicts of interest.

## Appendix Supplementary materials

Image, application 1

Image, application 2

Image, application 3

Image, application 4

Image, application 5

Image, application 6
