# Supplementary material for: The psychosis metabolic risk calculator (PsyMetRiC) for young people with psychosis: International external validation and site-specific recalibration in two independent European samples
Source: Lancet Reg Health Eur. 2022 Aug 19;22:100493. doi: 10.1016/j.lanepe.2022.100493 (PMC9418905; doi:10.1016/j.lanepe.2022.100493)
Supplement: Supplementary file 1 [file mmc1.docx]

**Captions for Supplementary Material**

Supplementary Methods

Supplementary Tables

Supplementary Figures

Completed TRIPOD Checklist

Abstract – French

Abstract - Spanish
