# Supplementary material for: The psychosis metabolic risk calculator (PsyMetRiC) for young people with psychosis: International external validation and site-specific recalibration in two independent European samples
Source: Lancet Reg Health Eur. 2022 Aug 19;22:100493. doi: 10.1016/j.lanepe.2022.100493 (PMC9418905; doi:10.1016/j.lanepe.2022.100493)
Supplement: Supplementary file 2 [file mmc2.docx]

# The Psychosis Metabolic Risk Calculator (PsyMetRiC) for Young People with Psychosis: International External Validation and Site-Specific Recalibration in Two Independent European Samples

Perry, Vandenberghe & Garrido-Torres *et al*

**Supplementary Methods**

**Comparisons Between British, Spanish and Swiss Populations**

See Supplementary Table 1 for a detailed comparison of key sociodemographic, economic and healthcare-related metrics between the overall British, Spanish and Swiss populations. In summary, all three countries offer (predominantly) universal healthcare funded through taxation, however the UK NHS is generally considered less divergent, is more centralized and allows in principle broader access to equitable care than the Swiss and Spanish health systems^1^. Despite this, the NHS fairs lower than EU averages in many key indicators of health care performance^1^. While health expenditure per person is similar in the UK and Spain, both spend a significantly lower proportion of GDP on healthcare than Switzerland, which also has a much higher per capita income and higher taxation than both the UK and Spain, despite having a much smaller population. Switzerland has a higher cost of living compared with the UK and Spain. Life expectancy is similar in Spain and the UK, but both are lower than in Switzerland. Spain scores lower on political stability and civil rights compared with the UK and Switzerland. The UK scores substantially lower in ethnic and linguistic fractionalization^2^ than Spain and Switzerland, but is similar in religious fractionalization. Spain has a substantially higher unemployment rate compared with the UK and Switzerland. Diets differ between these countries, with the Mediterranean culture potentially offering diet driven improved cardiovascular outcomes^3^. The degree of taxation on ‘unhealthy’ products varies between countries. For example, while all three countries impose a ‘sugar tax’ on sweetened drinks, this ranges from 8% in Switzerland, to 20% in UK and 21% in Spain. Taxation on alcohol is generally higher in the UK compared with Spain and Switzerland^4^. The prevalence of smoking is substantially higher in Switzerland and Spain than in the UK.

**Missing Data**The amount and structure of missing data in the remaining sample was assessed, and considered for multiple imputation using chained equations^5^. To examine the pattern of missing data in both samples, we plotted ‘missingness maps’ and matrices comparing missing values in predictor variables against eachother. (see below).

**PAFIP – Missingness Map**

Missingness maps help to visualize patterns in missing data (white bars) compared with observed data (blue bars) between variables. sexo = sex; TabacoBinario = smoking status; metAP = metabolically-active antpsychotic; BMI0 = baseline body mass index; Edad = age; trigli0 = triglycerides; hdl0 = high-density lipoprotein cholesterol.


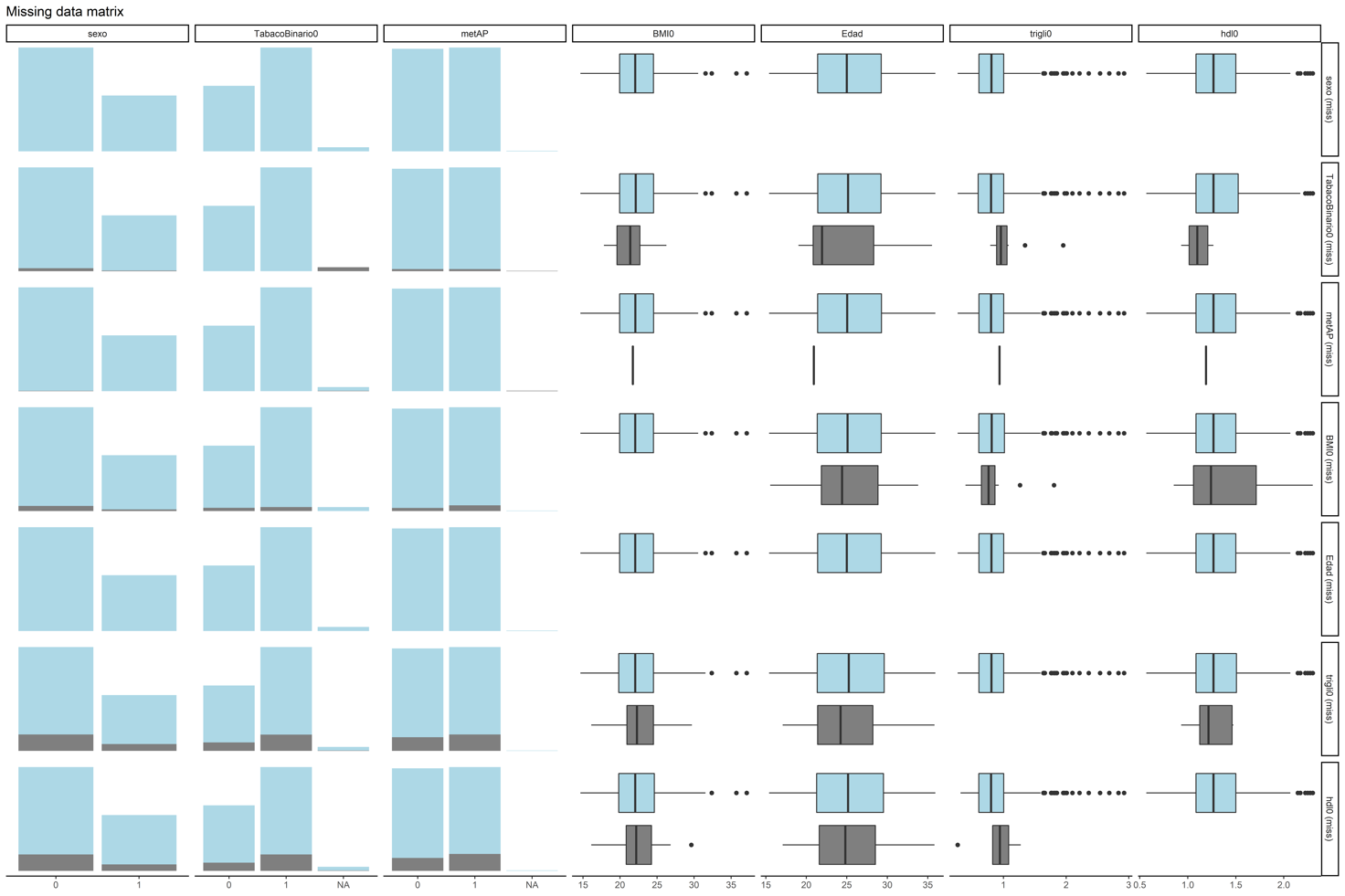
**PAFIP – Matrix of Missing Values Per Pair of Predictor Variables**

This plot shows a matrix of paired variables to show relationships between observed data (light blue) and missing data (grey). sexo = sex; TabacoBinario = smoking status; metAP = metabolically-active antpsychotic; BMI0 = baseline body mass index; Edad = age; trigli0 = triglycerides; hdl0 = high-density lipoprotein cholesterol.


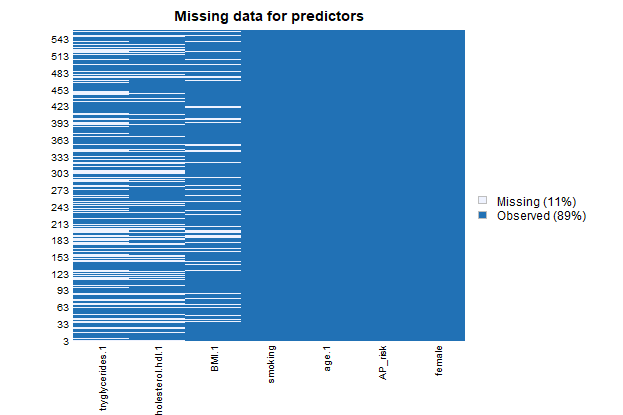
**PsyMetab – Missingness Map**

Missingness maps help to visualize patterns in missing data (white bars) compared with observed data (blue bars) between variables. sexo = sex; TabacoBinario = smoking status; metAP = metabolically-active antipsychotic; BMI0 = baseline body mass index; Edad = age; trigli0 = triglycerides; hdl0 = high-density lipoprotein cholesterol.


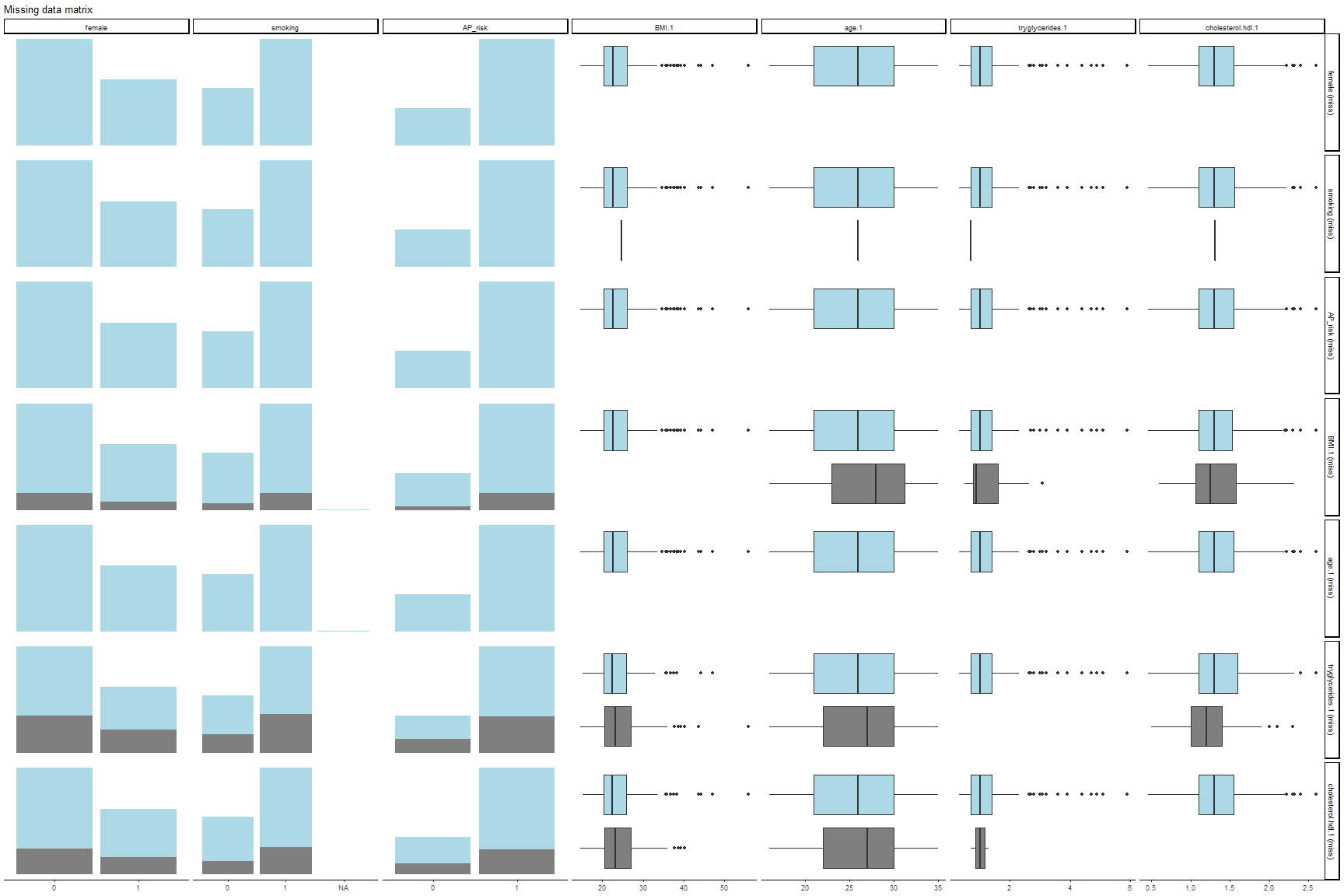
**PsyMetab – Matrix of Missing Values Per Predictor Variable**

This plot shows a matrix of paired variables to show relationships between observed data (light blue) and missing data (grey) between variables. female = sex; smoking = smoking status; AP_risk = metabolically-active antipsychotic; BMI.1 = baseline body mass index; age.1 = age; cholesterol.hdl = high-density lipoprotein cholesterol.

From the above missingness map plots, there appears a pattern of missing data such that when biochemical tests were not performed, all biochemical variables were missing together, as would be expected. There were no other common patterns of missing data between variables. In addition, from the matrices of missing values, there appears in both samples to be evidence of a weak association between sex and missingness for biochemical data (triglycerides and HDL) but no other clear patterns of missingness between variables. Therefore, we did not find strong evidence that the data were ‘missing not at random’. However, because some patterns of missing data were evident in our sample, we only considered variables for imputation where they were <50% missing^6^ and had suitable auxiliary variables available for use as indicators of missingness, in order to reduce the impact of ‘missing not at random’ bias^7^.

See the proportion of missing data per variable in each dataset below. We imputed 10 datasets in both samples. Box-and-Whisker and Density plots were used to check similarities of observed and imputed data. The MetS outcome was calculated from the constituent variables after the multiple imputation step.

| **Variable** | **PsyMetab** | **PAFIP** |
| --- | --- | --- |
| Sex | 0·00 | 0·00 |
| Ethnicity | 0·00 | 0·00 |
| Age | 0·00 | 0·00 |
| Smoking Status | <0·01 | 0·02 |
| Antipsychotic Prescription | 0·00 | <0·01 |
| SBP – Baseline | 0·24 | 0·24 |
| SBP – Follow-up | 0·23 | 0·47 |
| BMI – Baseline | 0·14 | 0·04 |
| BMI – Follow-up | 0·16 | 0·16 |
| Triglycerides – Baseline | 0·35 | 0·15 |
| Triglycerides – Follow-up | 0·27 | 0·14 |
| HDL – Baseline | 0·25 | 0·14 |
| HDL – Follow-up | 0·27 | 0·14 |

***Proportion of Missing Data Per Variable***

SBP = systolic blood pressure; BMI = body mass index; HDL = High-density lipoprotein cholesterol.

For numerical-based analyses, estimates were pooled using Rubin’s rules. For plot-based analyses, plots were generated in each imputed dataset and checked for similarity, with one randomly selected plot per analysis presented in the main manuscript and all remaining plots presented in the Supplementary Data.

**External Validation Analysis**The C-statistic is derived from the area under the curve and estimates the probability that a randomly selected ‘case’ will have a higher predicted probability for incident MetS than a randomly selected non-case. Scores of 1·0 indicate perfect discrimination; scores of 0·5 indicate that the algorithm is no better than chance; scores of >0·7 are generally considered acceptable^8^. Calibration plots estimate the accuracy of absolute-risk estimates (i.e. agreement between observed and predicted risk).

**Logistic Recalibration**

Logistic recalibration takes into account differences in baseline risk that may exist between populations by re-estimating the intercept term, and also re-estimates the slope term. Thus, logistic calibration assumes similar relative effects of the predictors but allows for larger or smaller absolute effects of the predictors^9^. To perform logistic recalibration, we fitted a regression equation with the linear predictor of the original regression equation as a single predictor in both samples separately. The algorithm was then updated in both samples separately by multiplying the linear predictor by the coefficient and adding the newly estimated intercept. The individual linear predictors and predicted probabilities for each participant were then recalculated and predictive performance was re-assessed.

**Clinical Usefulness and Potential Cutoffs**Decision curve analysis^10^ was used to assess the clinical usefulness of PsyMetRiC by estimating net benefit (Supplementary Methods). Net benefit is a metric of true positives minus false positives, and is calculated as: sensitivity × prevalence – (1 – specificity) × (1 – prevalence) × *w,*

where *w* is the outcome odds at a given risk threshold^11^. The risk threshold is the amount of tolerable risk before an intervention is deemed necessary. Net benefit incorporates the consequences of the decisions made on the basis of an algorithm, and is therefore preferable to related measures such as sensitivity and specificity alone^11^. We also reported the standardized net benefit (net benefit / outcome prevalence) and related metrics (sensitivity and specificity) across a range of reasonable risk thresholds. We drew a decision curve plot which visualised and compared the net benefit of the original PsyMetRiC algorithm vs the recalibrated country-specific version in each respective sample, compared with intervening in all, or intervening in none. Classical decision theory proposes that at a chosen risk-threshold, the choice with the greatest net-benefit should be preferred^11^.

### **Sample Size Power Calculation**

We used recently developed criteria^12^ to estimate the required sample size for an adequately powered validation study (Supplementary Methods). Since our study features retrospectively collected data with the available sample fixed, we used this calculation as a means to estimate the likely precision of our results. Briefly, the calculation is performed based on the anticipated outcome prevalence, the anticipated C-statistic, and the required standard errors (SEs) of the estimated C-statistic, calibration slope, and calibration-in-the-large. We obtained the anticipated outcome prevalence (16·86%) and C-statistic (0·75) from the external validation of the original PsyMetRiC study. Based on the available samples, the expected SEs for the C-statistic were 0·028 (Switzerland) and 0·029 (Spain). The expected SEs for the calibration slope and calibration-in-the-large were 0·14 & 0·13 (Switzerland), and 0·15 & 0·13 (Spain). For reference, for more precise^12^ SEs of the C-statistic (0·025), calibration slope (0·10) and calibration-in-the-large (0·10), a minimum sample size of *n=*1008 would be required.

**Analysis of Multi-Collinearity of Variables Included in PsyMetRiC in PsyMetab and PAFIP Samples**

| **Predictor** | **Variance Inflation Factor^a^** | |
| --- | --- | --- |
|  | **PsyMetab (Switzerland)** | **PAFIP (Spain)** |
| Sex | 1·16 | 1·15 |
| Asian/Other Ethnicity | 1·03 | 1·06 |
| Black African / Caribbean Ethnicity | 1·03 | 1·02 |
| Age | 1·02 | 1·24 |
| Body Mass Index | 1·05 | 1·10 |
| Current Smoking Status | 1·02 | 1·04 |
| Prescribed a More Metabolically-Active Antipsychotic | 1·05 | 1·03 |
| Triglycerides | 1·06 | 1·19 |
| HDL Cholesterol | 1·19 | 1·10 |

^a^Scores close to 1 indicate no evidence of multi-collinearity. Scores >5 indicate potential multi-collinearity.
^b^See Supplementary Table 4.

**References**

1. Robertson R. How Does the NHS Compare Internationally? Big Election Questions. The Kings Fund, 2017.

2. Fearon JD. Ethnic and Cultural Diversity by Country. *Journal of Economic Growth* 2003; **8**: 195-222.

3. Delgado-Lista J, Alcala-Diaz JF, Torres-Pena JD, et al. Long-term secondary prevention of cardiovascular disease with a Mediterranean diet and a low-fat diet (CORDIOPREV): a randomised controlled trial. *Lancet* 2022; **399**(10338): 1876-85.

4. Angus C, Holmes J, Meier PS. Comparing alcohol taxation throughout the European Union. *Addiction* 2019; **114**(8): 1489-94.

5. White IR, Royston P, Wood AM. Multiple imputation using chained equations: Issues and guidance for practice. *Stat Med* 2011; **30**(4): 377-99.

6. Lee HJ, Huber, J.,. Multiple imputation with large proportions of missing data: How much is too much? United Kingdom Stata Users' Group Meetings 2011; 2011: Stata Users Group; 2011.

7. Dong Y, Peng CY. Principled missing data methods for researchers. *Springerplus* 2013; **2**(1): 222.

8. Fukuma S, Shimizu S, Shintani A, Kamitani T, Akizawa T, Fukuhara S. Development and validation of a prediction model for loss of physical function in elderly hemodialysis patients. *Nephrol Dial Transplant* 2018; **33**(8): 1452-8.

9. Steyerberg EW, Borsboom GJ, van Houwelingen HC, Eijkemans MJ, Habbema JDF. Validation and updating of predictive logistic regression models: a study on sample size and shrinkage. *Statistics in medicine* 2004; **23**(16): 2567-86.

10. Vickers AJ, Elkin EB. Decision curve analysis: a novel method for evaluating prediction models. *Med Decis Making* 2006; **26**(6): 565-74.

11. Vickers AJ, van Calster B, Steyerberg EW. A simple, step-by-step guide to interpreting decision curve analysis. *Diagn Progn Res* 2019; **3**: 18.

12. Pavlou M, Qu C, Omar RZ, et al. Estimation of required sample size for external validation of risk models for binary outcomes. *Stat Methods Med Res* 2021; **30**(10): 2187-206.
