# Supplementary material for: The psychosis metabolic risk calculator (PsyMetRiC) for young people with psychosis: International external validation and site-specific recalibration in two independent European samples
Source: Lancet Reg Health Eur. 2022 Aug 19;22:100493. doi: 10.1016/j.lanepe.2022.100493 (PMC9418905; doi:10.1016/j.lanepe.2022.100493)
Supplement: Supplementary file 3 [file mmc3.docx]

# The Psychosis Metabolic Risk Calculator (PsyMetRiC) for Young People with Psychosis: International External Validation and Site-Specific Recalibration in Two Independent European Samples

Perry, Vandenberghe & Garrido-Torres *et al*

**Supplementary Tables**

**Supplementary Table 1: Key Comparison Metrics Between External Validation Samples and Original PsyMetRiC Development Sample**

| ***Category*** | ***Comparator*** | ***Country*** | | |
| --- | --- | --- | --- | --- |
|  |  | **United Kingdom** | **Switzerland** | **Spain** |
| **General** | Area (km^2^) | 243,610 | 41,290 | 505,935 |
|  | Government Form | Parliamentary Constitutional Monarchy | Federal Republic | Parliamentary Constitutional Monarchy |
| **Demographics** | Population, millions | 67·22 | 8·64 | 47·35 |
|  | Inhabitants/km^2^ | 275·92 | 209·23 | 93·60 |
|  | Life Expectancy (Males) (years) | 79 | 81 | 80 |
|  | Life Expectancy (Females) (years) | 83 | 85 | 85 |
|  | Quality of Life Score^1^ – Political Stability^2^ | 75 | 91 | 63 |
|  | Quality of Life Score^1^ – Civil Rights^3^ | 91 | 99 | 73 |
|  | Quality of Life Score^1^ – Health^4^ | 78 | 93 | 87 |
|  | Quality of Life Score^1^ – Cost of Living^5^ | 34 | 42 | 41 |
|  | Prevalence of Smoking (%)^6^ | 15·4 | 25·5 | 27·7 |
|  | Dominant Language (language, %) | English (97) | German (63) | Spanish (74) |
|  | Dominant Religion (religion, %) | Christianity (67) | Christianity (71) | Christianity (94) |
|  | Ethnic Fractionalization Score^7^ | 0·12 | 0·42 | 0·53 |
|  | Linguistic Fractionalization Score^7^ | 0·05 | 0·41 | 0·54 |
|  | Religious Fractionalization Score^7^ | 0·69 | 0·45 | 0·61 |
|  | Annual Net Migration per 1000 Inhabitants^8^ | 3·9 | 6·1 | 0·9 |
| **Economy** | Unemployment Rate (%) | 4·5 | 5·3 | 14·7 |
|  | Median income (USD) | 39,830 | 82,620 | 27,630 |
|  | Corruption Index^9^ | 78 | 84 | 61 |
|  | GDP per 1000 Inhabitants (USD, millions) | 41·06 | 87·10 | 27·06 |
| **Healthcare** | Hospital Beds per 1000 | 2·46 | 4·63 | 2·97 |
|  | Health Expenditure (%GDP)^10^ | 9·8 | 11·5 | 9·0 |
|  | Psychiatric Inpatient Beds per 1000^11^ | 0·46 | 0·91 | 0·36 |
|  | Medical Doctors per 1000 | 2·81 | 4·24 | 4·07 |
|  | T2D Hospitalization per 100,000^12^ | 59 | 48 | 50 |
|  | CHF Hospitalization per 100,000^12^ | 100 | 186 | 198 |
|  | CT Scanners per 1,000,000^12^ | 8·0 | 36·2 | 17·5 |

Information obtained from *worlddata.info* unless otherwise stated. ^1^scored from 0 (worst) – 100 (best). See <https://www.worlddata.info/quality-of-life.php> for more information on how scores were created. ^2^A metric comprising economic inflation, government debt/deficits, gross domestic product, unemployment rate, and ratio of available money supply to currency reserves. ^3^A metric comprising the regulatory quality of the government, democratic participation by populus, and the corruption index from *Transparency.org*. ^4^A metric comprising average life expectancy, drinking water supply, and the number of medical doctors and hospital beds. ^5^A metric comprising cost of living, median income, government taxation, and The World Bank ‘Ease of Doing Business’ index. ^6^Data obtained via *The World Bank* <https://data.worldbank.org>; ^7^Fractionalization scores are commonly used metrics in economics, and show the probability that two randomly drawn individuals from the population are not from the same group (e.g., ethnic, religious, linguistic). 2022 fractionalization scores were taken from *worldpopulationreview.com*. ^8^Data obtained from United Nations <https://population.un.org/>. ^9^based on scores from *Transparency International* Corruption Perceptions Index ([https://www.transparency.org/en/cpi/2021/index/](https://www.transparency.org/en/cpi/2021/index/usa)). ^10^Taken from OECD Health At a Glance: Europe 2016 Report [https://read.oecd-ilibrary.org/social-issues-migration-health/health-at-a-glance-europe-2016](https://read.oecd-ilibrary.org/social-issues-migration-health/health-at-a-glance-europe-2016_9789264265592-en#page185).  ^11^Taken from World Health Organization European Health Information Gateway. ^12^Taken from OECD Reviews of Health Care Quality 2016 Report [https://read.oecd-ilibrary.org/social-issues-migration-health/oecd-reviews-of-health-care-quality-united-kingdom-2016](https://read.oecd-ilibrary.org/social-issues-migration-health/oecd-reviews-of-health-care-quality-united-kingdom-2016_9789264239487-en#page116).
GDP=gross domestic product; T2D=type 2 diabetes; CHF=congestive heart failure.

**Supplementary Table 2: Diagnostic Classification of Included Participants**

| **Diagnosis** | **Sample** | |
| --- | --- | --- |
|  | **PsyMetab, *N* (%)** | **PAFIP, *N* (%)** |
| Psychotic Disorders^1^ | 317 (56·81) | 457 (98·07) |
| Schizoaffective Disorder^2^ | 44 (7·86) | 9 (1·97) |
| Bipolar Disorder with Psychotic Symptoms^3^ | 68 (12·19) | 0 (0) |
| Not Recorded^4^ | 129 (23·12) | 0 (0) |

^1^Psychotic disorders encompass ICD codes F20-F24, F28 and F29; ^2^Schizoaffective disorders encompass ICD codes F25; ^3^Bipolar disorders encompass ICD codes F30-F31; ^4^Diagnosis not recorded but participants included based on the presence of an antipsychotic prescription.

**Supplementary Table 3: Association of Individual PsyMetRiC Predictors with Metabolic Syndrome in the PsyMetab and PAFIP Samples**

| **PsyMetRiC Predictor** | **Effect Estimate^a^ (95% CI), *p*-value** | |
| --- | --- | --- |
|  | **PsyMetab** | **PAFIP** |
| Age | β = 0·05 (-0·01, 0·10), *p*=0·099 | β = 0·01 (-0·05, 0·07), *p*=0·772 |
| Black/African-Caribbean Ethnicity | OR = 0·85 (0·46, 1·24), *p*=0·690 | OR = 1·65 (0·85, 4·90), *p*=0·376 |
| Asian / Other Ethnicity | OR = 1·05 (0·59-1·51), *p*=0·909 | OR = 3·03 (0·90, 9·32), *p*=0·063 |
| Male Sex | OR = 1·21 (0·96-1·46), *p*=0·455 | OR = 2·01 (1·04, 3·98), *p*=0·044 |
| Body Mass Index (BMI) (kg/m^2^) | β = 0·11 (0·07-0·16), *p*<0·001 | β = 0·17 (0·09, 0·24), *p*<0·001 |
| Current Smoking Status | OR = 1·43 (0·87-2·44), *p*=0·232 | OR = 1·11 (0·48, 1·74), *p*=0·766 |
| Prescribed a Metabolically-Active Antipsychotic^b^ | OR = 1·21 (0·79-2·88), *p*=0·325 | OR = 1·05 (0·45, 1·66), *p=*0·867 |
| High-Density Lipoprotein (HDL) (mmol/L) | β = -2·31 (1·10, 3·42), *p*<0·001 | β = -2·90 (-4·10, -1·68), *p*<0·001 |
| Triglycerides (mmol/L) | β = 0·55 (0·25, 0·85), *p*<0·001 | β = 1·04 (0·39, 1·69), *p*=0·002 |

^a^For binary predictors, we present odds ratios estimated from univariable logistic regression analysis. For continuous predictors, we present beta coefficients estimated from univariable linear regression analysis

^b^See Supplementary Table 4

| **More Metabolically-Active Antipsychotics** | **Less Metabolically-Active Antipsychotics** |
| --- | --- |
| Olanzapine^1^* | Aripiprazole^1^* |
| Quetiapine^1^* | Amisulpiride^1^* |
| Risperidone^1^* | Haloperidol^1^ |
| Paliperidone^1^ | Sulpiride^2^ |
| Clozapine^1^ | Pericyazine^3†^ |
| Chlorpromazine^1^ | Lurasidone^1†^ |
| Asenapine^4†^ | Ziprasdone^1†^ |
| Pimozide^2†^ | Flupenthixol^4†^ |
| Levomepromazine^2†^ | Fluphenazine^4†^ |
| Prochlorperazine^6†^ | Zuclopenthixol^2†^ |
| Trifluoperazine^5†^ |  |
| Pipotiazine^5†^ |  |

**Supplementary Table 4: Classification of More Metabolically-Active Antipsychotics**

This table comprises all antipsychotics prescribed for participants/patients in all samples.
*indicates the five most commonly prescribed antipsychotics across all samples
^†^indicates antipsychotics rarely prescribed (<3 participants/patients in total across all samples)

**Supplementary Table 5: Results of Logistic Calibration of PsyMetRiC in PsyMetab and PAFIP Samples**

|  | **PsyMetab** | | **PAFIP** | |
| --- | --- | --- | --- | --- |
| **PsyMetRiC Version** | **New Intercept** | **New Slope** | **New Intercept** | **New Slope** |
| Full Model | -5·099451 | 0·7730436 | -6·226315 | 1·09828 |
| Partial Model | -6·582914 | 0·9365388 | -6·712295 | 0·911652 |

| **Measure of Predictive Performance** | **Primary Analysis, Estimate (99% C.I.)** | | **After Logistic Calibration, Estimate (99% C.I.)** | |
| --- | --- | --- | --- | --- |
|  | **Full-Model** | **Partial-Model** | **Full-Model** | **Partial-Model** |
| **PsyMetab (Switzerland)** | | | | |
| C-Statistic | 0·73 (0·64, 0·83) | 0·68 (0·59, 0·77) | 0·73 (0·64, 0·83) | 0·68 (0·59, 0·77) |
| r^2^ | 0·10 (0·05, 0·15) | 0·08 (0·02, 0·14) | 0·12 (0·07, 0·16) | 0·08 (0·03, 0·13) |
| Calibration Intercept | 0·11 (-0·06, 0·26) | 0·12 (0·03, 0·21) | -0·01 (-0·02, 0·00) | -0·01 (-0·02, 0·00) |
| Calibration Slope | 0·77 (0·70, 0·85) | 0·93 (0·83, 1·04) | 1·02 (1·00, 1·04) | 1·03 (1·00, 1·06) |
| Brier Score | 0·13 (0·08, 0·18) | 0·14 (0·05, 0·23) | 0·13 (0·06, 0·18) | 0·14 (0·09, 0·19) |
| **PAFIP (Spain)** | | | | |
| C-Statistic | 0·72 (0·63, 0·81) | 0·66 (0·57, 0·74) | 0·72 (0·63, 0·81) | 0·66 (0·57, 0·74) |
| r^2^ | 0·10 (0·04, 0·16) | 0·05 (0·01, 0·09) | 0·10 (0·04, 0·16) | 0·05 (0·01, 0·09) |
| Calibration Intercept | 0·24 (0·06, 0·40) | -0·30 (-0·41, -0·19) | 0·01 (-0·01, 0·02) | 0·01 (-0·01, 0·02) |
| Calibration Slope | 1·09 (0·94, 1·27) | 0·91 (0·85, 1·07) | 1·03 (0·98, 1·07) | 1·04 (0·98, 1·12) |
| Brier Score | 0·13 (0·05, 0·18) | 0·12 (0·06, 0·19) | 0·11 (0·05, 0·17) | 0·12 (0·07, 0·18) |

**Supplementary Table 6: Predictive Performance Statistics of PsyMetRiC Before and After Logistic Calibration in PsyMetab and PAFIP with 99% Confidence Intervals^a^**

^a^It is customary to present predictive performance estimates accompanied by 95% CIs (derived from an alpha-value of 0·05 as commonly used in inferential statistics). However, since analyses of the same model were conducted twice (before and after logistic calibration), we divided the 0·05 value by 2 (0·05/2=0·025) and thus present estimates alongside 99% confidence intervals as an additional sensitivity analysis. See Table 3 for the primary analysis results.

The C-statistic is a measure of discrimination and estimates the probability that a randomly selected ‘case’ will have a higher predicted probability than a randomly selected non-case. Scores of 1·0 indicate perfect discrimination; scores of >0·70 are generally considered acceptable. The calibration intercept (ideally close to 0) and calibration slope (ideally close to 1) are estimates of model calibration (i.e., the agreement between the observed proportion and predicted risk). The Brier score (ideally close to 0, with scores >0·25 indicating poor performance) is an overall measure of algorithm performance.

**Supplementary Table 7: Pooled Net Benefit and Standardized Net Benefit of The PsyMetRiC Full-Model (Original and Recalibrated Versions) in PsyMetab Across a Range of Feasible Risk Thresholds**

| **Risk Threshold^a^** | **Net Benefit (Recalibrated) + 95% CI** | **Net Benefit (Original) + 95% CI** | **Standardized Net Benefit (Recalibrated) + 95% CI^b^** | **Standardized Net Benefit (Original) + 95% CI^b^** |
| --- | --- | --- | --- | --- |
| 0·00 | 0·185 (0·147,0·213) | 0·185 (0·151,0·215) | 1 (1·000,1·000) | 1 (1·000,1·000) |
| 0·01 | 0·176 (0·138,0·205) | 0·176 (0·142,0·207) | 0·955 (0·941,0·963) | 0·955 (0·943,0·963) |
| 0·02 | 0·168 (0·13,0·197) | 0·168 (0·133,0·199) | 0·91 (0·882,0·925) | 0·91 (0·885,0·926) |
| 0·03 | 0·159 (0·121,0·189) | 0·16 (0·125,0·191) | 0·863 (0·82,0·886) | 0·866 (0·83,0·889) |
| 0·04 | 0·151 (0·112,0·181) | 0·153 (0·118,0·184) | 0·818 (0·761,0·849) | 0·827 (0·782,0·856) |
| 0·05 | 0·143 (0·103,0·173) | 0·145 (0·111,0·177) | 0·775 (0·703,0·813) | 0·786 (0·727,0·831) |
| 0·06 | 0·136 (0·096,0·166) | 0·133 (0·099,0·165) | 0·738 (0·654,0·783) | 0·72 (0·644,0·774) |
| 0·07 | 0·13 (0·091,0·161) | 0·128 (0·095,0·157) | 0·707 (0·603,0·756) | 0·691 (0·617,0·765) |
| 0·08 | 0·122 (0·08,0·152) | 0·127 (0·095,0·158) | 0·66 (0·551,0·716) | 0·688 (0·614,0·758) |
| 0·09 | 0·114 (0·074,0·143) | 0·116 (0·087,0·144) | 0·615 (0·498,0·683) | 0·628 (0·549,0·711) |
| 0·10 | 0·109 (0·07,0·138) | 0·111 (0·083,0·139) | 0·588 (0·472,0·656) | 0·6 (0·516,0·697) |
| 0·11 | 0·109 (0·069,0·139) | 0·105 (0·078,0·133) | 0·591 (0·464,0·664) | 0·569 (0·483,0·659) |
| 0·12 | 0·107 (0·068,0·138) | 0·088 (0·058,0·114) | 0·578 (0·442,0·653) | 0·476 (0·363,0·577) |
| 0·13 | 0·099 (0·063,0·129) | 0·08 (0·051,0·108) | 0·539 (0·407,0·624) | 0·435 (0·306,0·544) |
| 0·14 | 0·093 (0·055,0·122) | 0·077 (0·05,0·105) | 0·504 (0·348,0·595) | 0·415 (0·301,0·532) |
| 0·15 | 0·091 (0·052,0·12) | 0·072 (0·046,0·096) | 0·493 (0·351,0·584) | 0·389 (0·273,0·495) |
| 0·16 | 0·087 (0·049,0·115) | 0·062 (0·034,0·09) | 0·472 (0·329,0·562) | 0·335 (0·201,0·442) |
| 0·17 | 0·078 (0·042,0·104) | 0·061 (0·036,0·087) | 0·421 (0·276,0·521) | 0·331 (0·207,0·436) |
| 0·18 | 0·069 (0·036,0·093) | 0·06 (0·036,0·085) | 0·373 (0·231,0·465) | 0·325 (0·21,0·42) |
| 0·19 | 0·062 (0·031,0·085) | 0·055 (0·031,0·081) | 0·333 (0·206,0·43) | 0·299 (0·186,0·393) |
| 0·20 | 0·06 (0·031,0·084) | 0·053 (0·028,0·079) | 0·323 (0·189,0·426) | 0·286 (0·161,0·387) |
| 0·21 | 0·059 (0·028,0·084) | 0·046 (0·023,0·069) | 0·317 (0·181,0·421) | 0·251 (0·126,0·353) |
| 0·22 | 0·053 (0·024,0·078) | 0·044 (0·019,0·066) | 0·289 (0·152,0·398) | 0·236 (0·112,0·332) |
| 0·23 | 0·05 (0·024,0·073) | 0·041 (0·017,0·065) | 0·273 (0·146,0·374) | 0·225 (0·112,0·325) |
| 0·24 | 0·048 (0·022,0·071) | 0·033 (0·012,0·056) | 0·258 (0·126,0·359) | 0·18 (0·073,0·282) |
| 0·25 | 0·047 (0·022,0·07) | 0·033 (0·012,0·056) | 0·252 (0·129,0·349) | 0·178 (0·068,0·281) |
| 0·26 | 0·047 (0·023,0·069) | 0·032 (0·012,0·056) | 0·255 (0·136,0·348) | 0·174 (0·07,0·283) |
| 0·27 | 0·046 (0·021,0·068) | 0·029 (0·012,0·053) | 0·249 (0·123,0·344) | 0·158 (0·065,0·269) |
| 0·28 | 0·041 (0·016,0·063) | 0·029 (0·01,0·05) | 0·221 (0·098,0·325) | 0·155 (0·061,0·27) |
| 0·29 | 0·042 (0·017,0·06) | 0·028 (0·009,0·051) | 0·225 (0·105,0·326) | 0·15 (0·056,0·255) |

^a^Different risk thresholds may be selected depending on the proposed intervention (i.e., balancing the risk/benefit of exposing false positives to an intervention to benefit the most true positives), as well as patient or clinician preference.

^b^Standardized net benefit is calculated as the net benefit / outcome prevalence, showing the proportion of improvement in net benefit at the selected risk threshold.

**Supplementary Table 8: Pooled Net Benefit and Standardized Net Benefit of The PsyMetRiC Partial-Model (Original and Recalibrated Versions) in PsyMetab Across a Range of Feasible Risk Thresholds**

| **Risk Threshold^a^** | **Net Benefit (Recalibrated) + 95% CI** | **Net Benefit (Original) + 95% CI** | **Standardized Net Benefit (Recalibrated) + 95% CI^b^** | **Standardized Net Benefit (Original) + 95% CI^b^** |
| --- | --- | --- | --- | --- |
| 0·00 | 0·185 (0·154,0·21) | 0·185 (0·149,0·219) | 1 (1·000,1·000) | 1 (1·000,1·000) |
| 0·01 | 0·176 (0·146,0·202) | 0·176 (0·14,0·211) | 0·955 (0·945,0·962) | 0·955 (0·942,0·964) |
| 0·02 | 0·168 (0·137,0·194) | 0·168 (0·131,0·203) | 0·91 (0·888,0·923) | 0·91 (0·883,0·927) |
| 0·03 | 0·159 (0·128,0·185) | 0·159 (0·122,0·194) | 0·863 (0·83,0·883) | 0·863 (0·823,0·889) |
| 0·04 | 0·151 (0·119,0·177) | 0·151 (0·113,0·186) | 0·816 (0·771,0·843) | 0·816 (0·762,0·851) |
| 0·05 | 0·142 (0·11,0·168) | 0·142 (0·105,0·178) | 0·768 (0·711,0·802) | 0·769 (0·703,0·813) |
| 0·06 | 0·133 (0·101,0·16) | 0·133 (0·096,0·17) | 0·722 (0·655,0·764) | 0·723 (0·642,0·776) |
| 0·07 | 0·124 (0·09,0·152) | 0·124 (0·087,0·159) | 0·67 (0·587,0·722) | 0·672 (0·584,0·728) |
| 0·08 | 0·112 (0·076,0·139) | 0·112 (0·073,0·146) | 0·607 (0·497,0·67) | 0·606 (0·488,0·675) |
| 0·09 | 0·105 (0·069,0·134) | 0·104 (0·067,0·138) | 0·568 (0·44,0·638) | 0·562 (0·451,0·644) |
| 0·10 | 0·097 (0·06,0·126) | 0·097 (0·06,0·132) | 0·525 (0·388,0·602) | 0·526 (0·402,0·617) |
| 0·11 | 0·095 (0·058,0·124) | 0·091 (0·054,0·129) | 0·513 (0·378,0·592) | 0·493 (0·364,0·587) |
| 0·12 | 0·089 (0·054,0·117) | 0·087 (0·049,0·123) | 0·484 (0·356,0·569) | 0·47 (0·33,0·571) |
| 0·13 | 0·081 (0·047,0·106) | 0·084 (0·049,0·123) | 0·437 (0·302,0·528) | 0·456 (0·323,0·565) |
| 0·14 | 0·082 (0·048,0·109) | 0·072 (0·038,0·108) | 0·445 (0·311,0·536) | 0·392 (0·244,0·494) |
| 0·15 | 0·07 (0·038,0·095) | 0·072 (0·036,0·108) | 0·38 (0·251,0·49) | 0·388 (0·236,0·500) |
| 0·16 | 0·065 (0·033,0·088) | 0·069 (0·036,0·106) | 0·35 (0·211,0·452) | 0·375 (0·227,0·498) |
| 0·17 | 0·063 (0·035,0·087) | 0·06 (0·03,0·095) | 0·342 (0·216,0·454) | 0·324 (0·173,0·449) |
| 0·18 | 0·058 (0·032,0·081) | 0·053 (0·02,0·079) | 0·315 (0·189,0·416) | 0·288 (0·12,0·404) |
| 0·19 | 0·053 (0·025,0·075) | 0·049 (0·019,0·076) | 0·285 (0·156,0·389) | 0·263 (0·117,0·371) |
| 0·20 | 0·05 (0·023,0·072) | 0·047 (0·017,0·076) | 0·269 (0·145,0·38) | 0·257 (0·12,0·372) |
| 0·21 | 0·051 (0·025,0·073) | 0·046 (0·017,0·076) | 0·275 (0·146,0·371) | 0·25 (0·092,0·365) |
| 0·22 | 0·044 (0·019,0·064) | 0·042 (0·012,0·073) | 0·237 (0·108,0·335) | 0·228 (0·069,0·351) |
| 0·23 | 0·046 (0·02,0·064) | 0·038 (0·008,0·069) | 0·249 (0·124,0·346) | 0·208 (0·048,0·329) |
| 0·24 | 0·039 (0·014,0·058) | 0·039 (0·008,0·067) | 0·213 (0·078,0·306) | 0·209 (0·047,0·322) |
| 0·25 | 0·033 (0·009,0·054) | 0·038 (0·01,0·065) | 0·181 (0·051,0·27) | 0·207 (0·057,0·317) |
| 0·26 | 0·037 (0·013,0·056) | 0·037 (0·009,0·062) | 0·201 (0·078,0·288) | 0·198 (0·052,0·304) |
| 0·27 | 0·036 (0·013,0·052) | 0·032 (0·004,0·06) | 0·193 (0·08,0·274) | 0·175 (0·028,0·28) |
| 0·28 | 0·033 (0·01,0·05) | 0·035 (0·006,0·062) | 0·177 (0·059,0·258) | 0·19 (0·038,0·305) |
| 0·29 | 0·034 (0·012,0·051) | 0·029 (0·001,0·054) | 0·183 (0·076,0·265) | 0·159 (0·008,0·268) |
| 0·30 | 0·03 (0·01,0·048) | 0·023 (-0·003,0·048) | 0·162 (0·055,0·254) | 0·125 (-0·024,0·236) |

^a^Different risk thresholds may be selected depending on the proposed intervention (i.e., balancing the risk/benefit of exposing false positives to an intervention to benefit the most true positives), as well as patient or clinician preference.

^b^Standardized net benefit is calculated as the net benefit / outcome prevalence, showing the proportion of improvement in net benefit at the selected risk threshold.

**Supplementary Table 9: Pooled Net Benefit and Standardized Net Benefit of The PsyMetRiC Full-Model (Original and Recalibrated Versions) in PAFIP Across a Range of Feasible Risk Thresholds**

| **Risk Threshold^a^** | **Net Benefit (Recalibrated) + 95% CI** | **Net Benefit (Original) + 95% CI** | **Standardized Net Benefit (Recalibrated) + 95% CI^b^** | **Standardized Net Benefit (Original) + 95% CI^b^** |
| --- | --- | --- | --- | --- |
| 0·00 | 0·144 (0·114,0·174) | 0·144 (0·114,0·172) | 1 (1·000,1·000) | 1 (1·000,1·000) |
| 0·01 | 0·135 (0·105,0·165) | 0·135 (0·105,0·163) | 0·94 (0·921,0·952) | 0·94 (0·921,0·951) |
| 0·02 | 0·126 (0·096,0·157) | 0·126 (0·096,0·155) | 0·879 (0·842,0·904) | 0·879 (0·841,0·902) |
| 0·03 | 0·118 (0·087,0·149) | 0·118 (0·087,0·147) | 0·819 (0·764,0·856) | 0·82 (0·762,0·855) |
| 0·04 | 0·11 (0·08,0·141) | 0·109 (0·078,0·138) | 0·767 (0·695,0·813) | 0·761 (0·687,0·814) |
| 0·05 | 0·102 (0·071,0·134) | 0·106 (0·073,0·136) | 0·71 (0·624,0·775) | 0·735 (0·648,0·795) |
| 0·06 | 0·098 (0·067,0·13) | 0·086 (0·055,0·116) | 0·681 (0·578,0·75) | 0·601 (0·467,0·689) |
| 0·07 | 0·086 (0·055,0·116) | 0·08 (0·052,0·109) | 0·6 (0·482,0·683) | 0·554 (0·434,0·661) |
| 0·08 | 0·072 (0·041,0·102) | 0·073 (0·046,0·098) | 0·501 (0·369,0·6) | 0·508 (0·381,0·617) |
| 0·09 | 0·068 (0·039,0·096) | 0·063 (0·036,0·09) | 0·475 (0·353,0·57) | 0·438 (0·299,0·543) |
| 0·10 | 0·066 (0·038,0·092) | 0·052 (0·027,0·074) | 0·456 (0·325,0·553) | 0·365 (0·212,0·474) |
| 0·11 | 0·063 (0·035,0·088) | 0·045 (0·021,0·065) | 0·437 (0·307,0·541) | 0·313 (0·16,0·431) |
| 0·12 | 0·056 (0·032,0·081) | 0·039 (0·016,0·06) | 0·389 (0·254,0·495) | 0·273 (0·119,0·399) |
| 0·13 | 0·055 (0·031,0·079) | 0·035 (0·015,0·055) | 0·38 (0·244,0·485) | 0·241 (0·103,0·367) |
| 0·14 | 0·042 (0·02,0·069) | 0·035 (0·015,0·054) | 0·289 (0·157,0·403) | 0·241 (0·116,0·372) |
| 0·15 | 0·043 (0·021,0·071) | 0·027 (0·009,0·048) | 0·301 (0·166,0·423) | 0·19 (0·068,0·31) |
| 0·16 | 0·041 (0·018,0·068) | 0·025 (0·008,0·043) | 0·284 (0·153,0·413) | 0·171 (0·068,0·285) |
| 0·17 | 0·038 (0·015,0·065) | 0·023 (0·007,0·041) | 0·265 (0·117,0·398) | 0·16 (0·056,0·274) |
| 0·18 | 0·026 (0·002,0·053) | 0·021 (0·007,0·038) | 0·181 (0·017,0·315) | 0·146 (0·05,0·242) |
| 0·19 | 0·026 (0·003,0·054) | 0·017 (0·005,0·033) | 0·178 (0·021,0·314) | 0·121 (0·037,0·22) |
| 0·20 | 0·025 (0·002,0·053) | 0·011 (0·001,0·022) | 0·175 (0·016,0·309) | 0·075 (0·007,0·141) |
| 0·21 | 0·021 (0,0·047) | 0·011 (0·001,0·021) | 0·148 (-0·004,0·291) | 0·074 (0·011,0·143) |
| 0·22 | 0·019 (-0·003,0·043) | 0·012 (0·002,0·022) | 0·134 (-0·031,0·268) | 0·081 (0·018,0·148) |
| 0·23 | 0·021 (-0·002,0·046) | 0·009 (0·002,0·019) | 0·147 (-0·015,0·279) | 0·066 (0·011,0·133) |
| 0·24 | 0·019 (-0·002,0·042) | 0·007 (-0·001,0·016) | 0·132 (-0·018,0·261) | 0·05 (-0·011,0·107) |
| 0·25 | 0·019 (-0·001,0·04) | 0·008 (0,0·017) | 0·134 (-0·006,0·246) | 0·055 (0,0·116) |
| 0·26 | 0·018 (-0·002,0·039) | 0·006 (-0·001,0·012) | 0·128 (-0·019,0·241) | 0·04 (-0·007,0·081) |
| 0·27 | 0·018 (-0·002,0·037) | 0·003 (-0·002,0·01) | 0·126 (-0·013,0·224) | 0·024 (-0·01,0·063) |
| 0·28 | 0·02 (0,0·039) | 0·003 (-0·002,0·01) | 0·139 (0·003,0·238) | 0·024 (-0·011,0·063) |
| 0·29 | 0·012 (-0·005,0·028) | 0·001 (-0·002,0·006) | 0·086 (-0·041,0·178) | 0·009 (-0·015,0·04) |

^a^Different risk thresholds may be selected depending on the proposed intervention (i.e., balancing the risk/benefit of exposing false positives to an intervention to benefit the most true positives), as well as patient or clinician preference.

^b^Standardized net benefit is calculated as the net benefit / outcome prevalence, showing the proportion of improvement in net benefit at the selected risk threshold.

**Supplementary Table 10: Pooled Net Benefit and Standardized Net Benefit of The PsyMetRiC Partial-Model (Original and Recalibrated Versions) in PAFIP Across a Range of Feasible Risk Thresholds**

| **Risk Threshold^a^** | **Net Benefit (Recalibrated) + 95% CI** | **Net Benefit (Original) + 95% CI** | **Standardized Net Benefit (Recalibrated) + 95% CI^b^** | **Standardized Net Benefit (Original) + 95% CI^b^** |
| --- | --- | --- | --- | --- |
| 0·00 | 0·144 (0·114,0·178) | 0·144 (0·12,0·176) | 1 (1·000,1·000) | 1 (1·000,1·000) |
| 0·01 | 0·135 (0·105,0·17) | 0·135 (0·111,0·168) | 0·94 (0·921,0·953) | 0·94 (0·926,0·953) |
| 0·02 | 0·126 (0·096,0·161) | 0·126 (0·102,0·159) | 0·878 (0·841,0·906) | 0·878 (0·851,0·904) |
| 0·03 | 0·117 (0·086,0·153) | 0·117 (0·093,0·15) | 0·816 (0·759,0·857) | 0·816 (0·774,0·855) |
| 0·04 | 0·108 (0·077,0·144) | 0·108 (0·084,0·142) | 0·752 (0·677,0·809) | 0·752 (0·695,0·805) |
| 0·05 | 0·099 (0·068,0·136) | 0·099 (0·074,0·133) | 0·691 (0·597,0·765) | 0·69 (0·618,0·758) |
| 0·06 | 0·089 (0·059,0·127) | 0·09 (0·066,0·125) | 0·622 (0·511,0·712) | 0·628 (0·545,0·71) |
| 0·07 | 0·082 (0·053,0·12) | 0·08 (0·054,0·116) | 0·573 (0·453,0·675) | 0·558 (0·451,0·66) |
| 0·08 | 0·074 (0·043,0·112) | 0·073 (0·047,0·109) | 0·515 (0·386,0·629) | 0·51 (0·387,0·621) |
| 0·09 | 0·061 (0·033,0·096) | 0·065 (0·04,0·104) | 0·424 (0·295,0·536) | 0·45 (0·335,0·589) |
| 0·10 | 0·05 (0·023,0·082) | 0·054 (0·028,0·094) | 0·347 (0·186,0·474) | 0·376 (0·235,0·53) |
| 0·11 | 0·043 (0·016,0·076) | 0·045 (0·02,0·08) | 0·296 (0·129,0·432) | 0·312 (0·16,0·479) |
| 0·12 | 0·036 (0·011,0·066) | 0·034 (0·005,0·067) | 0·25 (0·089,0·406) | 0·234 (0·045,0·399) |
| 0·13 | 0·035 (0·009,0·068) | 0·031 (0·005,0·062) | 0·245 (0·081,0·397) | 0·213 (0·039,0·371) |
| 0·14 | 0·029 (0·005,0·057) | 0·026 (0·001,0·058) | 0·2 (0·043,0·342) | 0·183 (0·005,0·339) |
| 0·15 | 0·033 (0·007,0·062) | 0·025 (0·002,0·057) | 0·231 (0·065,0·377) | 0·176 (0·016,0·335) |
| 0·16 | 0·033 (0·011,0·061) | 0·018 (-0·004,0·049) | 0·23 (0·087,0·377) | 0·127 (-0·036,0·292) |
| 0·17 | 0·027 (0·007,0·054) | 0·02 (-0·001,0·05) | 0·191 (0·054,0·334) | 0·14 (-0·007,0·299) |
| 0·18 | 0·024 (0·005,0·049) | 0·024 (0·002,0·054) | 0·167 (0·039,0·318) | 0·169 (0·015,0·329) |
| 0·19 | 0·019 (0·002,0·043) | 0·021 (0·001,0·051) | 0·135 (0·015,0·282) | 0·149 (0·004,0·31) |
| 0·20 | 0·019 (0,0·041) | 0·018 (-0·005,0·045) | 0·131 (0,0·258) | 0·123 (-0·036,0·276) |
| 0·21 | 0·017 (-0·001,0·038) | 0·016 (-0·005,0·044) | 0·115 (-0·008,0·244) | 0·112 (-0·035,0·267) |
| 0·22 | 0·018 (0,0·04) | 0·013 (-0·007,0·039) | 0·128 (0·001,0·26) | 0·091 (-0·063,0·246) |
| 0·23 | 0·016 (0,0·036) | 0·012 (-0·008,0·034) | 0·113 (-0·002,0·245) | 0·085 (-0·07,0·22) |
| 0·24 | 0·013 (-0·002,0·031) | 0·012 (-0·008,0·035) | 0·092 (-0·012,0·186) | 0·083 (-0·056,0·218) |
| 0·25 | 0·01 (-0·004,0·027) | 0·012 (-0·007,0·035) | 0·07 (-0·033,0·176) | 0·085 (-0·048,0·215) |
| 0·26 | 0·011 (-0·002,0·028) | 0·017 (0,0·039) | 0·079 (-0·016,0·182) | 0·119 (-0·002,0·248) |
| 0·27 | 0·006 (-0·009,0·021) | 0·011 (-0·005,0·029) | 0·044 (-0·065,0·147) | 0·077 (-0·034,0·192) |
| 0·28 | 0·005 (-0·01,0·02) | 0·01 (-0·007,0·026) | 0·036 (-0·069,0·124) | 0·067 (-0·059,0·169) |
| 0·29 | 0·005 (-0·01,0·019) | 0·007 (-0·008,0·025) | 0·037 (-0·066,0·129) | 0·048 (-0·059,0·174) |
| 0·30 | 0 (0,0) | 0·008 (-0·006,0·024) | 0 (0,0) | 0·058 (-0·045,0·153) |

^a^Different risk thresholds may be selected depending on the proposed intervention (i.e., balancing the risk/benefit of exposing false positives to an intervention to benefit the most true positives), as well as patient or clinician preference.

^b^Standardized net benefit is calculated as the net benefit / outcome prevalence, showing the proportion of improvement in net benefit at the selected risk threshold.

**Supplementary Table 11: Missing Sample Comparison for PsyMetab**

| **Characteristic** | **Included Sample** | **Missing Sample** |
| --- | --- | --- |
| Final sample size, *N*. | 558 | 2294 |
| Age in Years, mean (SD) | 25·9 (5·32) | 47·56 (19·15) |
| White European/NA Ethnicity, *N*. (%) | 446 (79·93) | 2038 (88·84) |
| Black/African-Caribbean Ethnicity, *N*. (%) | 68 (12·19) | 124 (5·41) |
| Asian/Other Ethnicity, *N*. (%) | 44 (7·48) | 132 (5·75) |
| Male Sex, *N*. (%) | 345 (61·83) | 1082 (47·17) |
| HDL at baseline, mmol/L, mean (SD) | 1·33 (0·36) | 1·39 (0·43) |
| Triglycerides at baseline, mmol/L, mean (SD) | 1·16 (0·70) | 1·47 (1·06) |
| BMI at baseline, kg/m^2^, mean (SD) | 23·63 (5·00) | 25·34 (5·60) |
| FPG at baseline (mmol/L), mean (SD) | 4·95 (0·82) | 5·24 (1·18) |
| Systolic BP at baseline (mmHg), mean (SD) | 121 (14·00) | 124 (17·00) |
| Metabolically-Active psychotropic medication ^b^, *N*. (%) | 413 (74·01) | 1848 (80·55) |
| Smoking at baseline, *N*. (%) | 362 (64·87) | 1090 (47·52) |
| Follow-up time, years, mean (SD) | 2·48 (1·40) | 2·66 (1·57) |
| Antipsychotic Naïve at baseline, *N.* (%) | 361 (64·70) | 1663 (72·49) |
| Metabolic Syndrome at baseline, *N*. (%^c^) | 36 (6·06) | 325 (14·17) |
| Metabolic Syndrome at Follow-up, *N*. (%) | 103 (18·54) | 843 (36·74) |

HDL=high-density lipoprotein cholesterol; SD=standard deviation; BMI=body mass index; FPG=fasting plasma glucose; BP=blood pressure.

**Supplementary Table 12: Missing Sample Comparison for PAFIP**

| **Characteristic** | **Included Sample** | **Missing Sample** |
| --- | --- | --- |
| Final sample size, *N*. | 466 | 419 |
| Age in Years, mean (SD) | 25·5 (4·99) | 35·8 (10·29) |
| White European/NA Ethnicity, *N*. (%) | 435 (93·34) | 412 (98·33) |
| Black/African-Caribbean Ethnicity, *N*. (%) | 15 (3·22) | 1 (0·23) |
| Asian/Other Ethnicity, *N*. (%) | 16 (3·43) | 6 (1·43) |
| Male Sex, *N*. (%) | 303 (65·16) | 203 (48·44) |
| HDL at baseline, mmol/L, mean (SD) | 1·32 (0·34) | 1·44 (0·44) |
| Triglycerides at baseline, mmol/L, mean (SD) | 0·878 (0·40) | 1·08 (0·59) |
| BMI at baseline, kg/m^2^, mean (SD) | 22·50 (3·36) | 24·7 (4·48) |
| FPG at baseline (mmol/L), mean (SD) | 4·69 (0·55) | 4·84 (0·99) |
| Systolic BP at baseline (mmHg), mean (SD) | 119 (14·10) | 113 (16·00) |
| Metabolically-Active psychotropic medication ^b^, *N*. (%) | 234 (50·21) | 92 (21·96) |
| Smoking at baseline, *N*. (%) | 279 (59·90) | 219 (52·23) |
| Follow-up time, years, mean (SD) | 2·59 (0·73) | 2·71 (0·63) |
| Antipsychotic Naïve at baseline, *N.* (%) | 433 (92·92) | 396 (94·51) |
| Metabolic Syndrome at baseline, *N*. (%^c^) | 31 (6·24) | 31 (6·89) |

HDL=high-density lipoprotein cholesterol; SD=standard deviation; BMI=body mass index; FPG=fasting plasma glucose; BP=blood pressure.

**Supplementary Table 13: Sociodemographic Characteristics of Antipsychotic Naïve Sub-Sample in PsyMetab Compared With Main Analytic Sample**

| **Characteristic** | **Main Analytic Sample** | **Antipsychotic Naïve Sub-Sample** |
| --- | --- | --- |
| Final sample size, *N*. | 558 | 361 |
| Age in Years, mean (SD) | 25·9 (5·32) | 26·0 (5·35) |
| White European/NA Ethnicity, *N*. (%) | 446 (79·93) | 294 (81·44) |
| Black/African-Caribbean Ethnicity, *N*. (%) | 68 (12·19) | 34 (9·42) |
| Asian/Other Ethnicity, *N*. (%) | 44 (7·48) | 33 (9·14) |
| Male Sex, *N*. (%) | 345 (61·83) | 215 (59·56) |
| HDL at baseline, mmol/L, mean (SD) | 1·33 (0·36) | 1·36 (0·38) |
| Triglycerides at baseline, mmol/L, mean (SD) | 1·16 (0·70) | 1·17 (0·72) |
| BMI at baseline, kg/m^2^, mean (SD) | 23·63 (5·00) | 23·3 (4·92) |
| FPG at baseline (mmol/L), mean (SD) | 4·95 (0·82) | 4·91 (0·77) |
| Systolic BP at baseline (mmHg), mean (SD) | 121 (14·00) | 120 (14·01) |
| Metabolically-Active psychotropic medication ^b^, *N*. (%) | 413 (74·01) | 282 (78·11) |
| Smoking at baseline, *N*. (%) | 362 (64·87) | 234 (64·82) |
| Follow-up time, years, mean (SD) | 2·48 (1·40) | 2·40 (1·29) |
| Metabolic Syndrome at Follow-up, *N*. (%) | 103 (18·54) | 61 (16·90) |

HDL=high-density lipoprotein cholesterol; SD=standard deviation; BMI=body mass index; FPG=fasting plasma glucose; BP=blood pressure.

**Supplementary Table 14: Sociodemographic Characteristics of Antipsychotic Naïve Sub-Sample in PAFIP Compared With Main Analytic Sample**

| **Characteristic** | **Main Analytic Sample** | **Antipsychotic Naïve Sub-Sample** |
| --- | --- | --- |
| Final sample size, *N*. | 466 | 433 |
| Age in Years, mean (SD) | 25·5 (4·99) | 25·5 (5·02) |
| White European/NA Ethnicity, *N*. (%) | 435 (93·34) | 403 (93·07) |
| Black/African-Caribbean Ethnicity, *N*. (%) | 15 (3·22) | 14 (3·23) |
| Asian/Other Ethnicity, *N*. (%) | 16 (3·43) | 16 (3·70) |
| Male Sex, *N*. (%) | 303 (65·16) | 282 (65·13) |
| HDL at baseline, mmol/L, mean (SD) | 1·32 (0·34) | 1·32 (0·34) |
| Triglycerides at baseline, mmol/L, mean (SD) | 0·878 (0·40) | 0·873 (0·40) |
| BMI at baseline, kg/m^2^, mean (SD) | 22·50 (3·36) | 22·5 (3·36) |
| FPG at baseline (mmol/L), mean (SD) | 4·69 (0·55) | 4·70 (0·56) |
| Systolic BP at baseline (mmHg), mean (SD) | 119 (14·10) | 119 (14·01) |
| Metabolically-Active psychotropic medication ^b^, *N*. (%) | 234 (50·21) | 223 (51·50) |
| Smoking at baseline, *N*. (%) | 279 (59·90) | 260 (60·04) |
| Follow-up time, years, mean (SD) | 2·59 (0·73) | 2·59 (0·72) |
| Metabolic Syndrome at Follow-up, *N.* (%) | 66 (14·16) | 57 (13·16) |

HDL=high-density lipoprotein cholesterol; SD=standard deviation; BMI=body mass index; FPG=fasting plasma glucose; BP=blood pressure.

**References**

1. Leucht S, Cipriani A, Spineli L, et al. Comparative efficacy and tolerability of 15 antipsychotic drugs in schizophrenia: a multiple-treatments meta-analysis. *Lancet* 2013; **382**(9896): 951-62.

2. Bak M, Fransen A, Janssen J, van Os J, Drukker M. Almost all antipsychotics result in weight gain: a meta-analysis. *PLoS One* 2014; **9**(4): e94112.

3. Matar HE, Almerie MQ, Makhoul S, Xia J, Humphreys P. Pericyazine for schizophrenia. *Cochrane Database Syst Rev* 2014; (5): CD007479.

4. Pillinger T, McCutcheon RA, Vano L, et al. Comparative effects of 18 antipsychotics on metabolic function in patients with schizophrenia, predictors of metabolic dysregulation, and association with psychopathology: a systematic review and network meta-analysis. *Lancet Psychiatry* 2020; **7**(1): 64-77.

5. Alonso-Pedrero L, Bes-Rastrollo M, Marti A. Effects of antidepressant and antipsychotic use on weight gain: A systematic review. *Obes Rev* 2019; **20**(12): 1680-90.
