# Supplementary material for: The psychosis metabolic risk calculator (PsyMetRiC) for young people with psychosis: International external validation and site-specific recalibration in two independent European samples
Source: Lancet Reg Health Eur. 2022 Aug 19;22:100493. doi: 10.1016/j.lanepe.2022.100493 (PMC9418905; doi:10.1016/j.lanepe.2022.100493)
Supplement: Supplementary file 4 [file mmc4.docx]

# The Psychosis Metabolic Risk Calculator (PsyMetRiC) for Young People with Psychosis: International External Validation and Site-Specific Recalibration in Two Independent European Samples

Perry, Vandenberghe & Garrido-Torres *et al*

**Supplementary Figures**

**Supplementary Figure 1: Flow-Chart of Participants Included In The Study**

**B=PAFIP**

**A=PsyMetab**

Total Sample

*n* = 885

Total Sample

*n* = 2,852

Baseline Age >16 and ≤35

*n* = 659

Baseline Age >16 and ≤35

*n* = 1,129

With Psychosis-Spectrum Disorder Diagnoses

*n* = 474

With Psychosis-Spectrum Disorder Diagnoses

*n* = 969

Follow-up between 1-6 years

*n* = 474

Follow-up between 1-6 years

*n* = 594

Without Metabolic Syndrome at Baseline

*n* = 466

Without Metabolic Syndrome at Baseline

*n* = 558

**Supplementary Figure 2: Pooled Predicted Probabilities for PsyMetRiC in The PsyMetab Sample**

A

B

Predicted Probability

Predicted Probability

A = Full-Model (before recalibration); B = Partial-Model (before recalibration); C = Full-Model (after recalibration);
D = Partial-Model (after recalibration)

C

D

Predicted Probability

Predicted Probability

**Supplementary Figure 3: Calibration Plots Across Imputed Datasets for PsyMetRiC in The PsyMetab Sample**

**A = Full-Model**


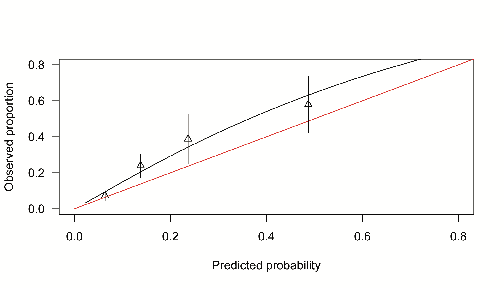

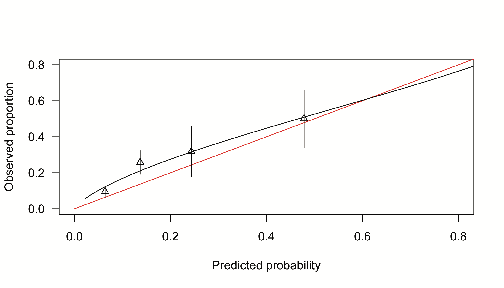

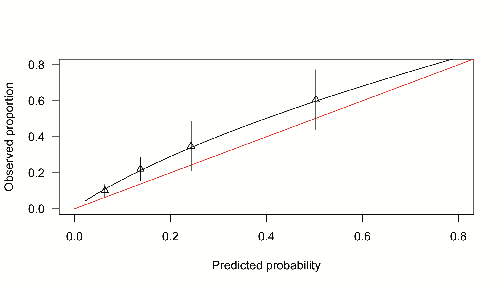

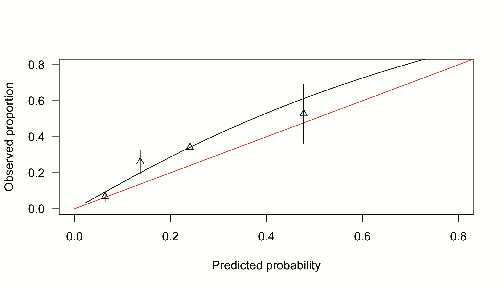

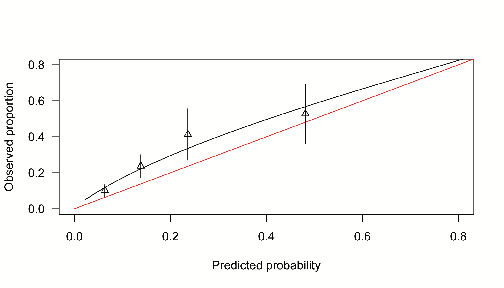

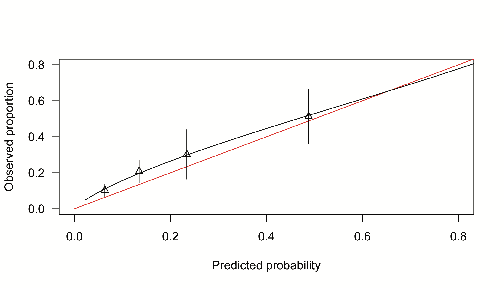

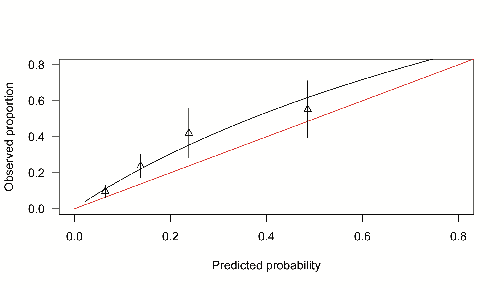

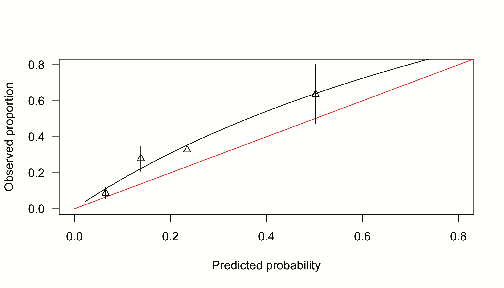

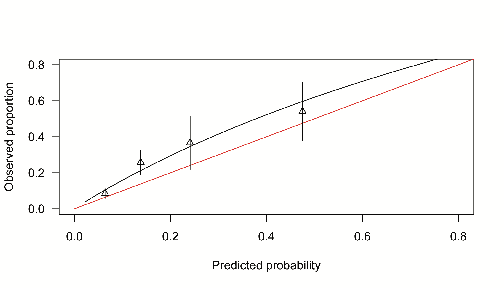


**B = Partial-Model**


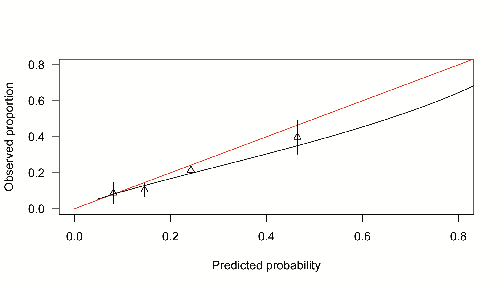

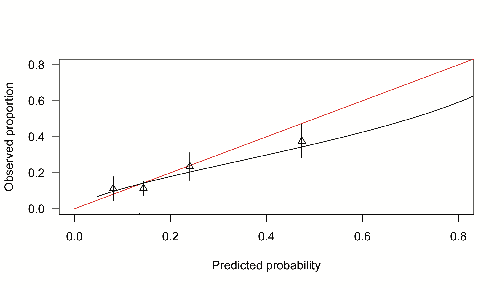

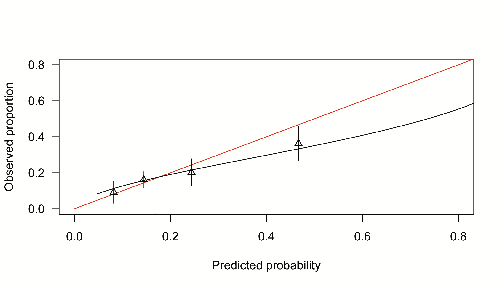

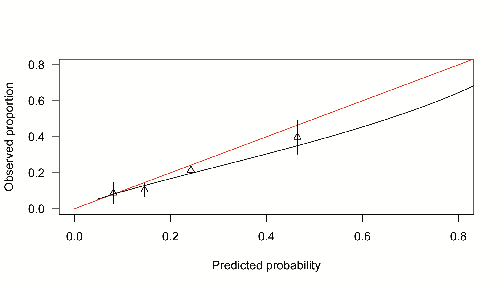

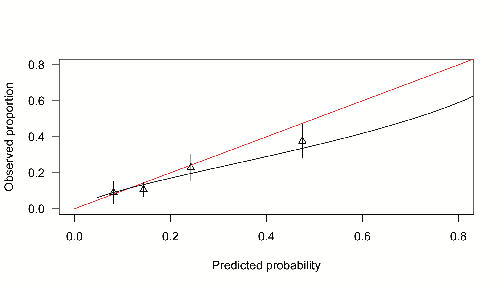

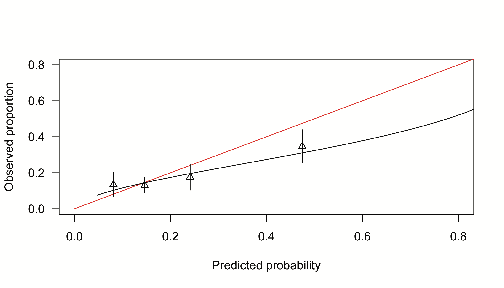

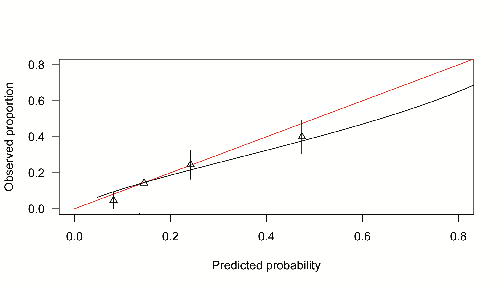

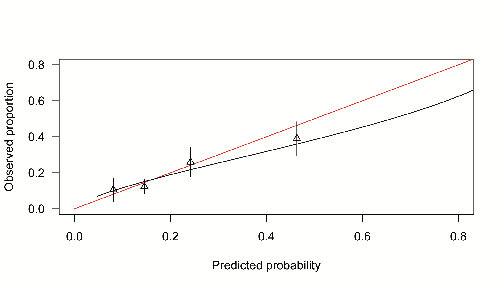

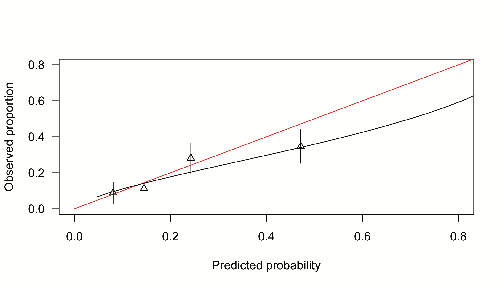


Calibration plots illustrate agreement between observed risk (y axis) and predicted risk (x axis). Perfect agreement would trace the red line. Algorithm calibration is illustrated by the black line. Triangles denote grouped observations for participants at deciles of predicted risk, with 95% C.I.’s indicated by the vertical black lines.

**Supplementary Figure 4: Pooled Predicted Probabilities for PsyMetRiC in The PAFIP Sample**

A

B

Predicted Probability

Predicted Probability

A = Full-Model (before recalibration); B = Partial-Model (before recalibration); C = Full-Model (after recalibration);
D = Partial-Model (after recalibration)

C

D

Predicted Probability

Predicted Probability

**Supplementary Figure 5: Calibration Plots Across Imputed Datasets for PsyMetRiC in The PAFIP Sample**

**A = Full-Model**


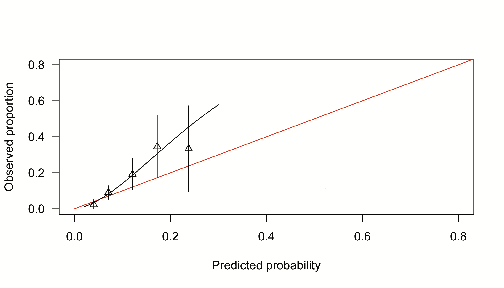

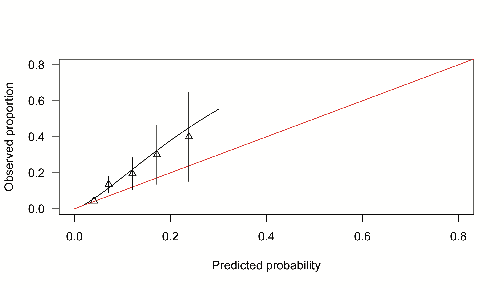

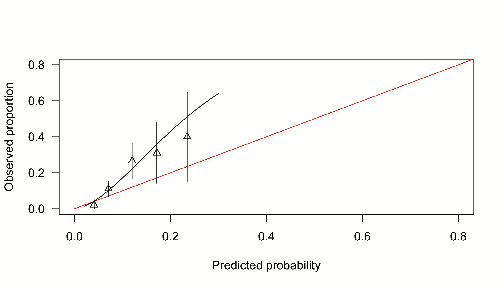

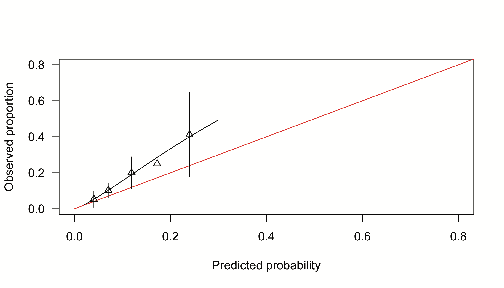

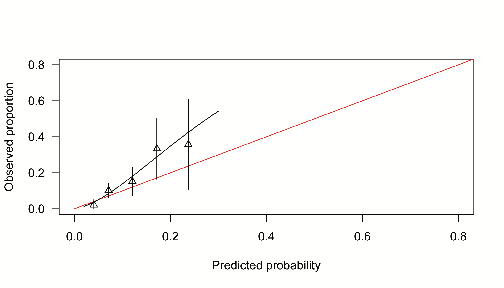

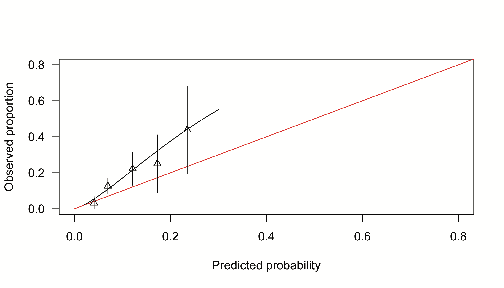

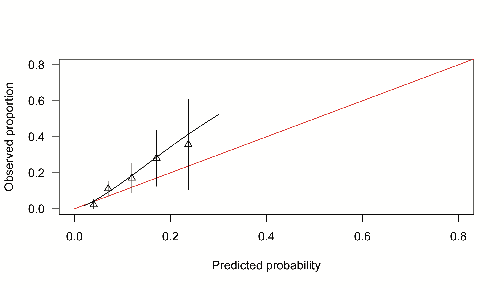

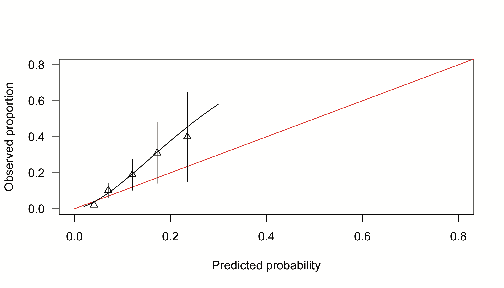

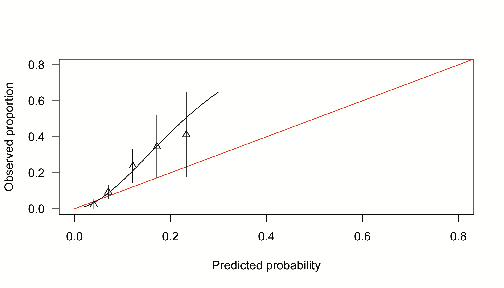


**B = Partial Model**


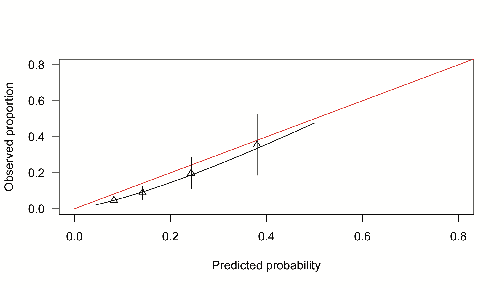

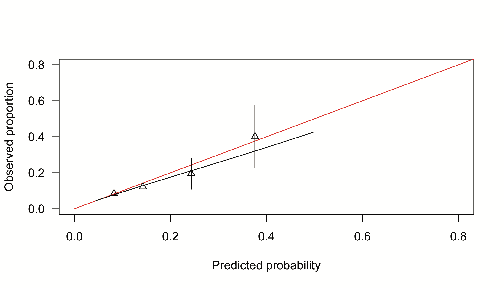

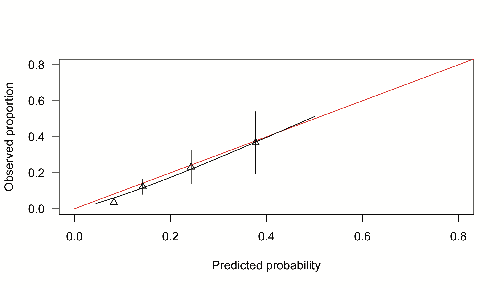

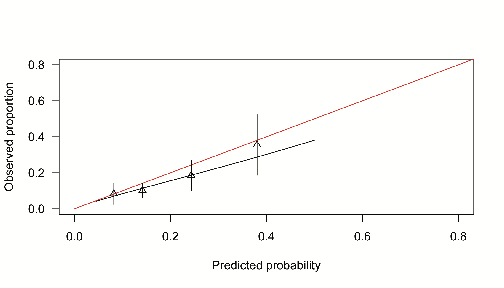

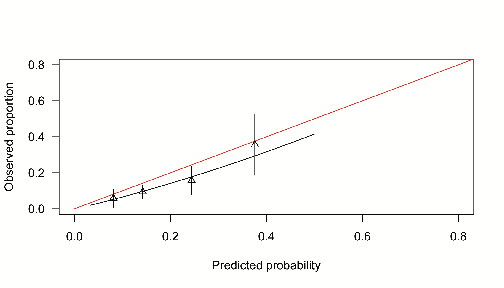

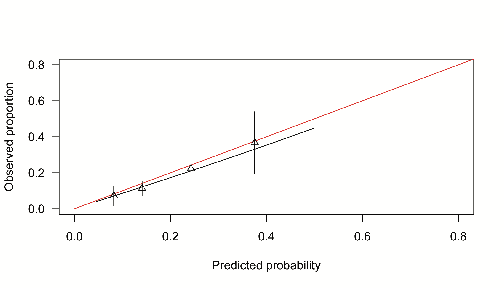

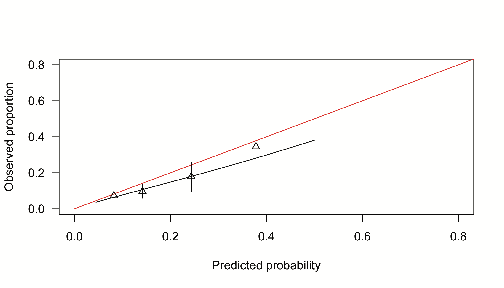

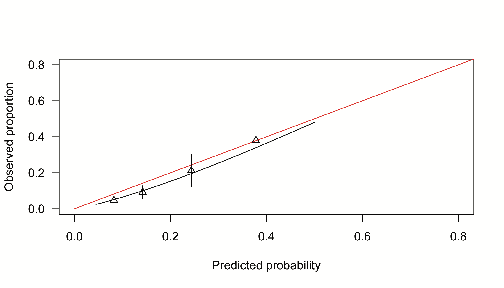

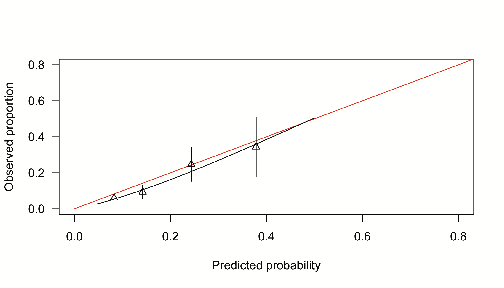


Calibration plots illustrate agreement between observed risk (y axis) and predicted risk (x axis). Perfect agreement would trace the red line. Algorithm calibration is illustrated by the black line. Triangles denote grouped observations for participants at deciles of predicted risk, with 95% C.I.’s indicated by the vertical black lines.

**Supplementary Figure 6: Calibration Plots Across Imputed Datasets for PsyMetRiC in The PsyMetab Sample After Logistic Calibration**

**A = Full-Model**


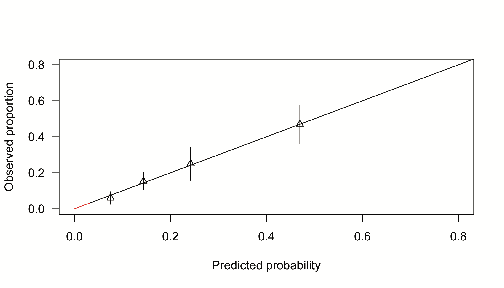

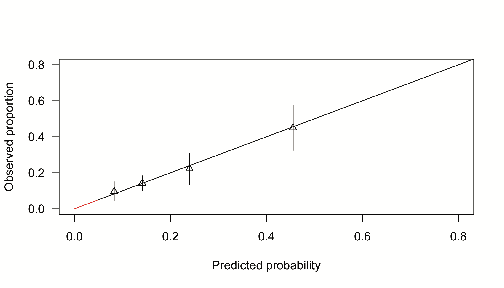

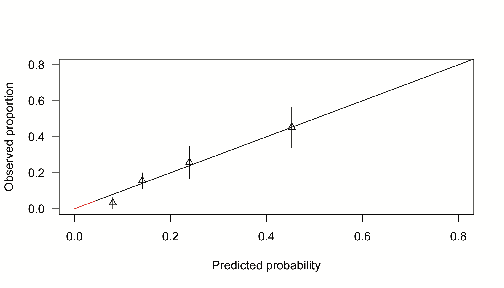

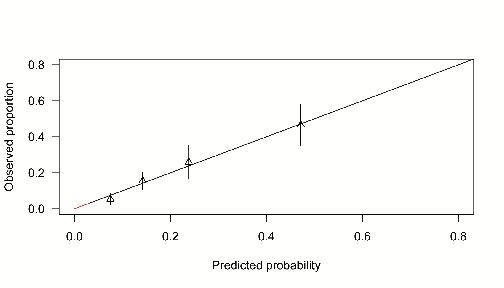

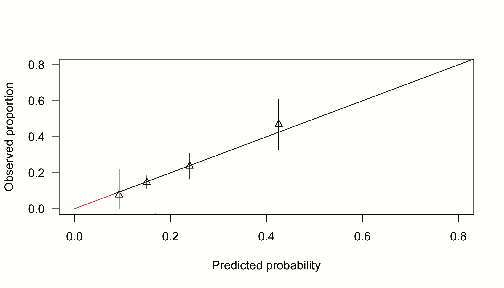

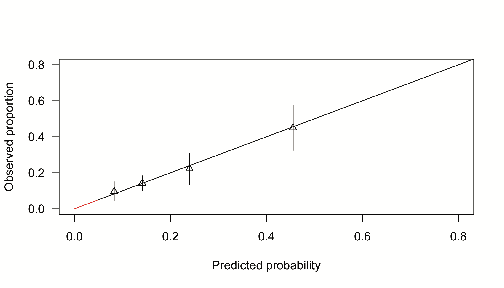

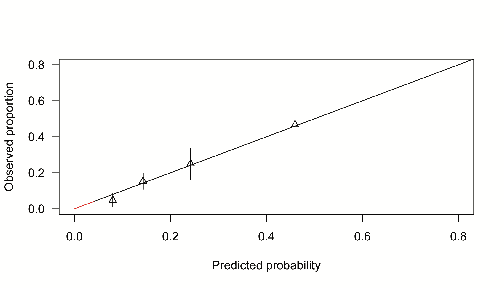

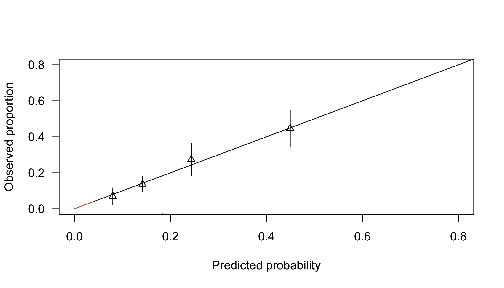

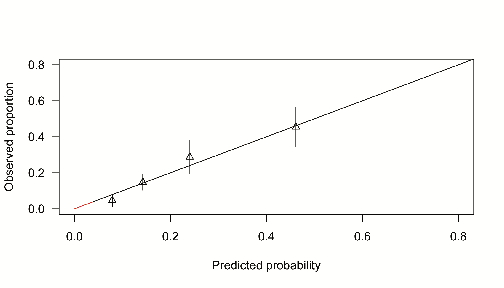


**B = Partial-Model**


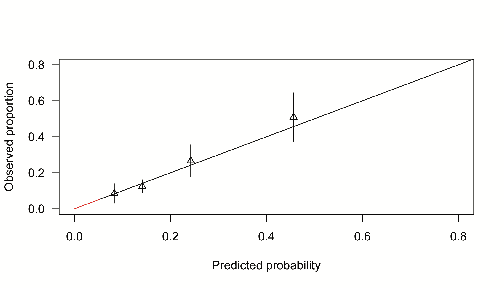

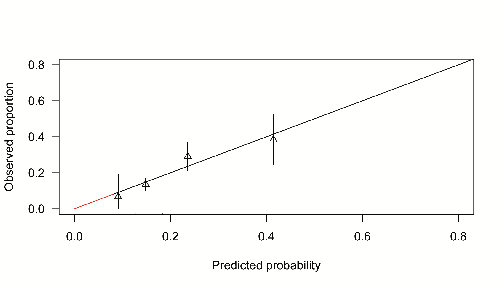

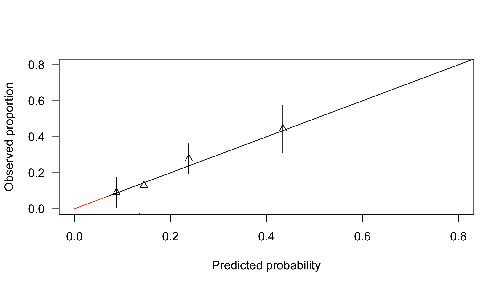

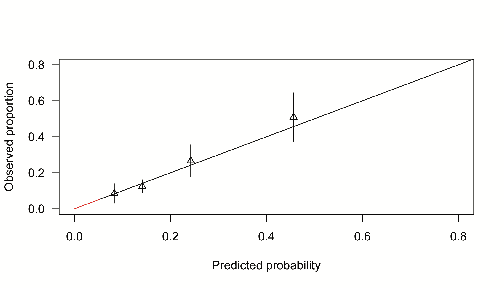

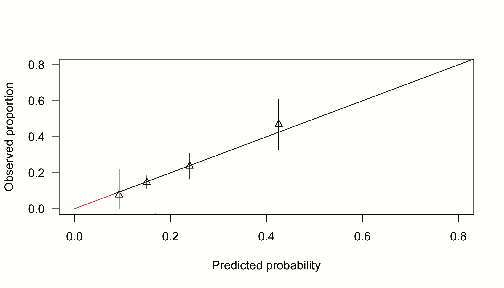

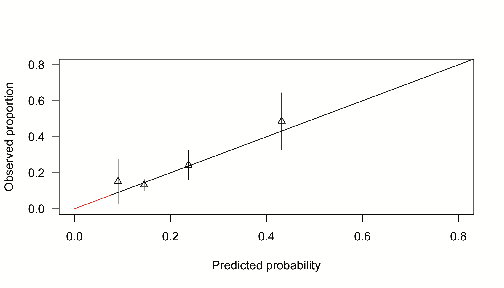

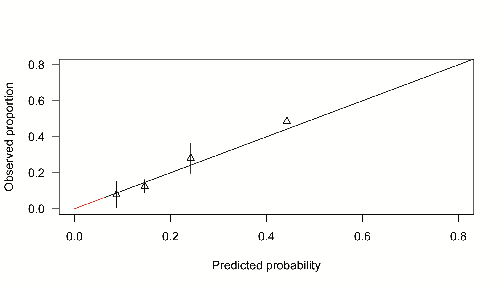

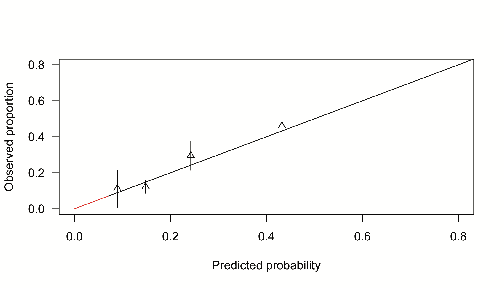

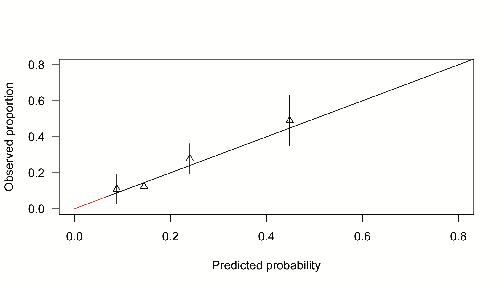


Calibration plots illustrate agreement between observed risk (y axis) and predicted risk (x axis). Perfect agreement would trace the red line. Algorithm calibration is illustrated by the black line. Triangles denote grouped observations for participants at deciles of predicted risk, with 95% C.I.’s indicated by the vertical black lines.

**Supplementary Figure 7: Calibration Plots Across Imputed Datasets for PsyMetRiC in The PAFIP Sample After Logistic Calibration**

**A = Full-Model**


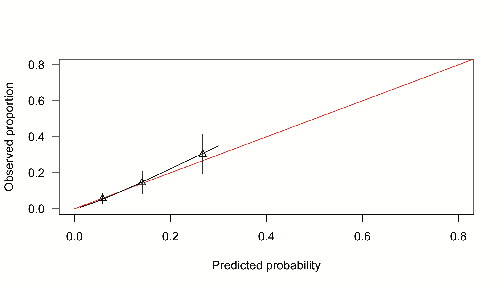

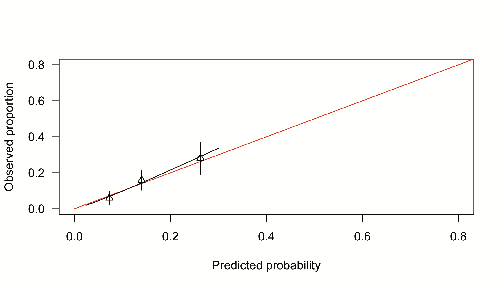

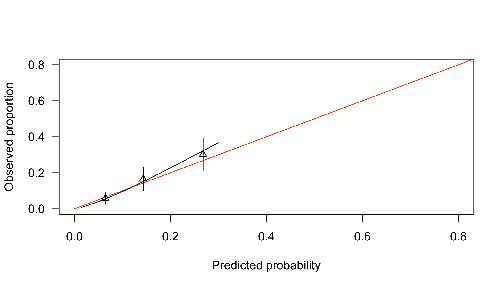

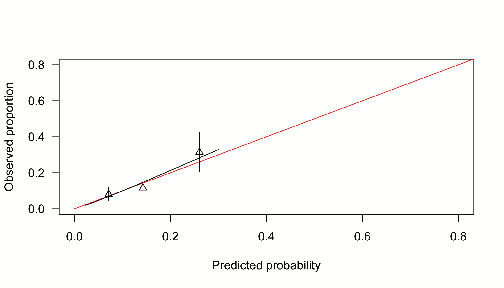

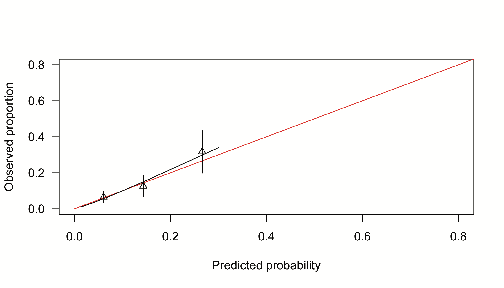

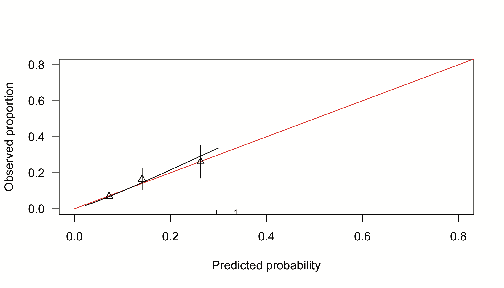

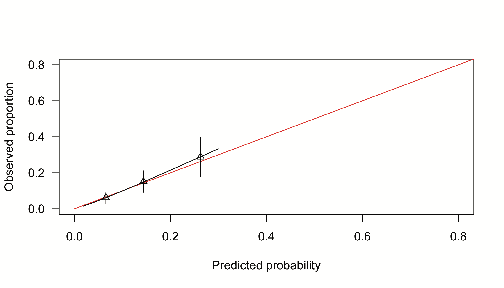

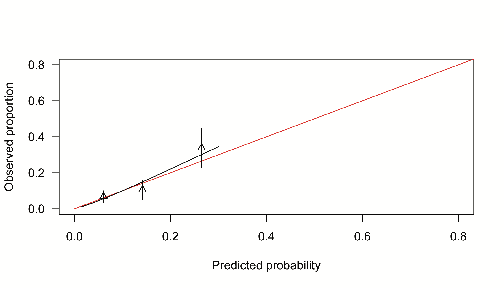

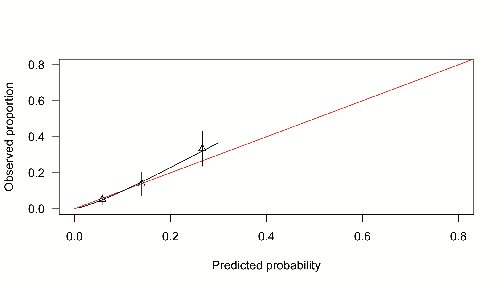


**B = Partial-Model**


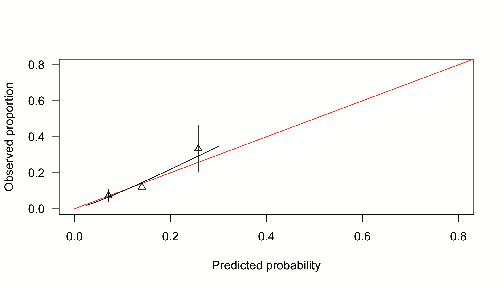

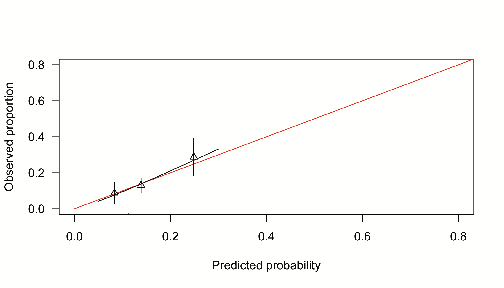

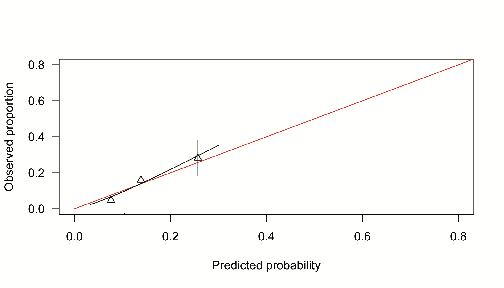

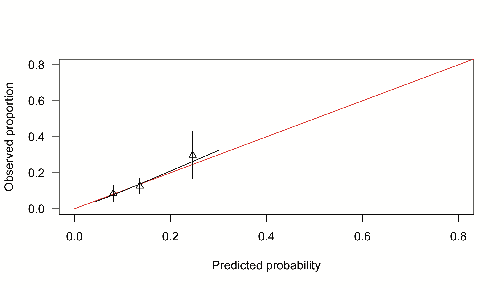

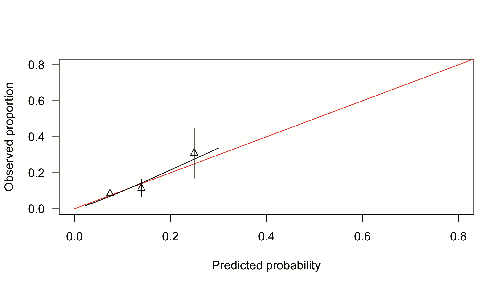

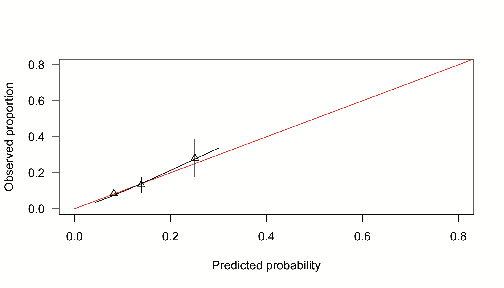

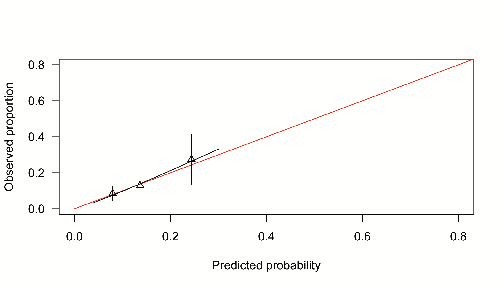

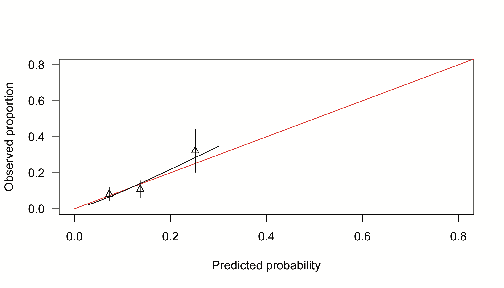

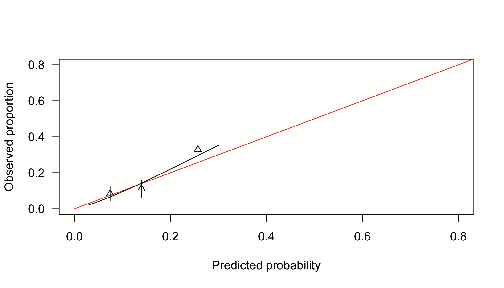


Calibration plots illustrate agreement between observed risk (y axis) and predicted risk (x axis). Perfect agreement would trace the red line. Algorithm calibration is illustrated by the black line. Triangles denote grouped observations for participants at deciles of predicted risk, with 95% C.I.’s indicated by the vertical black lines.

**Supplementary Figure 8: Clinical Usefulness Across Imputed Datasets of PsyMetRiC in The PsyMetab and PAFIP Samples Before and After Logistic Calibration**

**A = PsyMetab (Full-Model)**

**B = PsyMetab (Partial-Model)**

The plots report net benefit (y axis) of PsyMetRiC Full- and Partial-Models (original = blue line; recalibrated = red line) across a range of risk thresholds (x axis) compared with intervening in all (grey line) or intervening in none (black line).

**C = PAFIP (Full-Model)**

**D = PAFIP (Partial-Model)**

The plots report net benefit (y axis) of PsyMetRiC Full- and Partial-Models (original = blue line; recalibrated = red line) across a range of risk thresholds (x axis) compared with intervening in all (grey line) or intervening in none (black line).

**Supplementary Figure 9: Calibration Plots of PsyMetRiC Full- and Partial-Models When Tested in Subsamples of Antipsychotic Naïve Participants in PsyMetab and PAFIP**

A

B

C

D

A = Full-Model (PsyMetab); B = Partial-Model (PsyMetab); C = Full-Model (PAFIP); D = Partial-Model (PAFIP)

NB: Sensitivity analysis of antipsychotic naïve participants was conducted in all ten imputed datasets. Plots for other imputed datasets were similar to eachother as in the main analytic sample (see Supplementary Figures 4-7), and are available from the authors on request.

**Supplementary Figure 10: Simulated Case Scenarios to Visualize Impact of Modifiable and Non-Modifiable Risk Factors on Cardiometabolic Risk in Young People with Psychosis using Recalibrated Versions of PsyMetRiC (PsyMetRiC-CH and PsyMetRiC-ES)**

1. **PsyMetRiC-CH Full-Model**

A 24-year-old male of White European heritage is admitted to a psychiatric inpatient unit in Lausanne, Switzerland and diagnosed with psychosis. His BMI is toward the upper limit of the recommended range (24·7). He does not smoke. His blood test results for cholesterol are abnormal and suggest the possibility of insulin resistance^a^ (triglycerides = 2·51 mmol/L; HDL=1·03 mmol/L).

Initial PsyMetRiC-CH Score = 0·15

He is commenced on olanzapine.

New PsyMetRiC Score = 0·21

*40% increase in risk of metabolic syndrome*

He is commenced on aripiprazole.

.

New PsyMetRiC-CH Score = 0·15
*no change in risk of metabolic syndrome*

In time, he recovers from the acute psychotic episode but shows residual symptoms and so opts to remain on antipsychotic medication. He also accepts referral to a dietician to address his cholesterol levels. One year later (age 25y), a repeat blood test shows improvement: triglycerides=1·54mmol/L; HDL=1·33mmol/L and his BMI has decreased to 23·3. Due to residual symptoms of psychosis, his doctor talks with him about a possible change in medication.

He switches to olanzapine.

New PsyMetRiC-CH Score = 0·13

*13% decrease in risk of metabolic syndrome*

He continues on aripiprazole.

New PsyMetRiC-CH Score = 0·09

*40% decrease in risk of metabolic syndrome*

1. **PsyMetRiC-ES Partial-Model**

A 27-year-old South Asian female is diagnosed with psychosis in the community and enrolled in her local EIS in Cantabria, Spain. She accepts basic physical assessment only (BMI=26·2, in the ‘overweight’ range). She smokes 15 cigarettes per day.

Initial PsyMetRiC-ES Score = 0·12

She is commenced on risperidone.

New PsyMetRiC-ES Score = 0·19

*58% increase in risk of metabolic syndrome*

After she begins to recover from her psychotic symptoms, she is offered and commits to smoking cessation therapy and is successful in her efforts. She also talks to her doctor about a change in medication due to mild adverse effects.

She continues on risperidone.

New PsyMetRiC-ES Score = 0·15

*21% decrease in risk of metabolic syndrome*

She switches to amisulpride.

New PsyMetRiC-ES Score = 0·09

*52% decrease in risk of metabolic syndrome*

Along with some friends, she also joins a local sports club, and over the course of 1 year, her BMI has decreased to 24·3.

She is prescribed risperidone.

New PsyMetRiC-ES Score = 0·12

*20% decrease in risk of metabolic syndrome*

She is prescribed amisulpride.

New PsyMetRiC-ES Score = 0·07

*22% decrease in risk of metabolic syndrome*

PsyMetRiC scores presented as predicted probabilities, which can be converted to %chance of incident metabolic syndrome by multiplying by 100. ^a^A raised triglyceride:HDL ratio is indicative of insulin resistance
EIS=psychosis early intervention service; BMI=body mass index; HDL=high-density lipoprotein. The recalibrated PsyMetRiC versions for Switzerland and Spain require external validation and should not yet be used in clinical practice.
