# Supplementary material for: The psychosis metabolic risk calculator (PsyMetRiC) for young people with psychosis: International external validation and site-specific recalibration in two independent European samples
Source: Lancet Reg Health Eur. 2022 Aug 19;22:100493. doi: 10.1016/j.lanepe.2022.100493 (PMC9418905; doi:10.1016/j.lanepe.2022.100493)
Supplement: Supplementary file 5 [file mmc5.docx]

*This translation in French was submitted by the authors and we reproduce it as supplied. It has not been peer reviewed. Our editorial processes have only been applied to the original abstract in English, which should serve as reference for this manuscript.*

**Abstract (Français)**

**Contexte**Les troubles cardiométaboliques sont fréquents chez les jeunes atteints de psychose. Récemment, un outil (*Psychosis Metabolic Risk Calculator*; PsyMetRiC), permettant de prédire jusqu’à six ans le risque de survenue de syndrome métabolique (SMet) a été développé et validé au Royaume-Uni. Ce modèle inclu l'âge, le sexe, l'ethnicité, l'indice de masse corporelle, le statut tabagique, la prescription d'antipsychotiques et le profil lipidique (lipoprotéines de haute densité et triglycérides). Un modèle partiel a été développé en excluant le profil lipidique. Afin d'utiliser PsyMetRiC dans d'autres populations, nous avons entrepris une validation externe sur deux cohortes européennes indépendantes.

**Méthodes**Les données des cohortes PsyMetab (Lausanne, Suisse) et PAFIP (Cantabrie, Espagne), incluant des participants âgés de 16 à 35 ans sans SMet et avec un suivi prospectif de 1 à 6 ans ont été utilisées. La performance prédictive du modèle a été évaluée par discrimination (AUC=aire sous la courbe ROC), courbes de calibration et analyse des courbes de décision. Un recalibrage du modèle est envisagé si jugé nécessaire.

**Résultats**1,024 participants (PsyMetab n = 558, homme = 62 %, prévalence de MetS à la fin du suivi = 19 %, durée moyen du suivi = 2·48 ans ; PAFIP n = 466, homme = 65 %, prévalence de MetS à la fin du suivi = 14 %, durée moyen du suivi =2·59 ans) ont été inclus. La discrimination était meilleure dans le modèle complet en comparaison avec le modèle partiel (PsyMetab=modèle complet AUC=0·73, IC à 95 %, 0·68-0·79, modèle partiel AUC=0·68, IC à 95 %, 0·62-0·74 ; PAFIP=modèle complet AUC=0·72, IC à 95 %, 0·66-0·78 ; modèle partiel AUC=0·66, IC à 95 %, 0·60-0·71). Comme prévu, les courbes de calibration ont révélé des erreurs de classification, qui se sont rétablis après un recalibrage en fonction des deux cohortes. PsyMetRiC a permis de mettre en avant un bénéfice net dans les deux nouvelles cohortes, ce de manière encore plus évidente après le recalibrage.

**Interprétation**Cette analyse permet la généralisation de PsyMetRiC en Europe occidentale, bien que d'autres études de validation locales et internationales soient nécessaires. À l'avenir, PsyMetRiC pourrait aider les cliniciens à identifier les jeunes adultes atteints de psychose à risque cardiométabolique élevé, ouvrant ainsi la voie à des interventions individualisées dans le but de réduire la morbidité et la mortalité à long terme.

**Financement**

NIHR Cambridge Biomedical Research Centre (BRC-1215-20014); The Wellcome Trust (201486/Z/16/Z); Swiss National Research Foundation (320030-120686, 324730- 144064, and 320030-173211); The Carlos III Health Institute (CM20/00015, FIS00/3095, PI020499, PI050427, and PI060507); IDIVAL (INT/A21/10 and INT/A20/04); The Andalusian Regional Government (A1-0055-2020 and A1-0005-2021); SENY Fundacion Research (2005-0308007); Fundacion Marques de Valdecilla (A/02/07, API07/011); Ministry of Economy and Competitiveness and the European Fund for Regional Development (SAF2016-76046-R and SAF2013-46292-R).
