# Supplementary material for: The psychosis metabolic risk calculator (PsyMetRiC) for young people with psychosis: International external validation and site-specific recalibration in two independent European samples
Source: Lancet Reg Health Eur. 2022 Aug 19;22:100493. doi: 10.1016/j.lanepe.2022.100493 (PMC9418905; doi:10.1016/j.lanepe.2022.100493)
Supplement: Supplementary file 6 [file mmc6.docx]

*This translation in Spanish was submitted by the authors and we reproduce it as supplied. It has not been peer reviewed. Our editorial processes have only been applied to the original abstract in English, which should serve as reference for this manuscript.*

**Abstract (Español)**

**Antecedentes**Las alteraciones cardiometabólicas son comunes en los pacientes jóvenes que presentan psicosis. Recientemente, en el Reino Unido se desarrolló y validó externamente la Calculadora de riesgo metabólico de psicosis (PsyMetRiC), la cual ha demostrado predecir hasta seis años de riesgo de síndrome metabólico (MetS) a partir de datos clínicos recopilados de forma rutinaria. El modelo completo incluye: edad, sexo, origen étnico, índice de masa corporal, tabaquismo, prescripción de medicamentos antipsicóticos metabólicamente activos, lipoproteínas de alta densidad y triglicéridos. El modelo parcial excluye predictores bioquímicos.

**Métodos**Para avanzar hacia una futura herramienta de utilidad internacional, validamos externamente PsyMetRiC en dos muestras europeas independientes. Utilizamos datos de las cohortes PsyMetab (Lausana, Suiza) y PAFIP (Cantabria, España), se incluyeron participantes de 16 a 35 años que no presentaban MetS al inicio y se hizo un seguimiento de 1 a 6 años. El rendimiento predictivo se evaluó principalmente a través de la discriminación (estadística C), la calibración (gráficos de calibración) y el análisis de la curva de decisión. Se consideró además la recalibración específica de cada sitio.

**Recomendaciones**Se incluyeron 1024 participantes (PsyMetab n = 558, hombres = 62 %, prevalencia= 19 %, seguimiento medio = 2 · 48 años; PAFIP n = 466, hombres = 65 %, prevalencia = 14 %, seguimiento medio =2·59 años). La discriminación fue mejor en el modelo completo en comparación con el modelo parcial (PsyMetab = modelo completo C=0,73, IC del 95 %, 0,68-0,79, modelo parcial C=0,68, IC del 95 %, 0 ·62-0·74; PAFIP=modelo completo C=0·72, 95% IC, 0·66-0·78; modelo parcial C=0·66, 95% IC, 0·60-0·71 ). Como era de esperar, los plots de calibración revelaron diversos grados de mala calibración, que se recuperaron después de la recalibración específica. PsyMetRiC mostró un beneficio neto en ambas cohortes nuevas, más aún después de la recalibración.

**Interpretación**El estudio proporciona evidencia de la generalización de PsyMetRiC en Europa occidental, aunque se requieren más estudios de validación locales e internacionales. En el futuro, PsyMetRiC podría ayudar a los médicos a nivel internacional a identificar a los pacientes jóvenes psicosis que presenten mayor riesgo cardiometabólico, de esta forma las intervenciones podrían dirigirse de manera efectiva para reducir la morbilidad y la mortalidad a largo plazo.

**Financiamiento**

NIHR Cambridge Biomedical Research Centre (BRC-1215-20014); The Wellcome Trust (201486/Z/16/Z); Swiss National Research Foundation (320030-120686, 324730- 144064, and 320030-173211); The Carlos III Health Institute (CM20/00015, FIS00/3095, PI020499, PI050427, and PI060507); IDIVAL (INT/A21/10 and INT/A20/04); The Andalusian Regional Government (A1-0055-2020 and A1-0005-2021); SENY Fundacion Research (2005-0308007); Fundacion Marques de Valdecilla (A/02/07, API07/011); Ministry of Economy and Competitiveness and the European Fund for Regional Development (SAF2016-76046-R and SAF2013-46292-R).
